# Whole‐genome analyses provide no evidence for dog introgression in Fennoscandian wolf populations

**DOI:** 10.1111/eva.13151

**Published:** 2020-11-09

**Authors:** Linnéa Smeds, Jouni Aspi, Jonas Berglund, Ilpo Kojola, Konstantin Tirronen, Hans Ellegren

**Affiliations:** ^1^ Department of Ecology and Genetics Uppsala University Uppsala Sweden; ^2^ Department of Ecology and Genetics University of Oulu Oulu Finland; ^3^ Natural Resources Institute Finland (Luke) Rovaniemi Finland; ^4^ Institute of Biology Karelian Research Centre of the Russian Academy of Science Petrozavodsk Russian Federation

**Keywords:** admixture, conservation genomics, grey wolf, hybridization, introgression

## Abstract

Hybridization and admixture can threaten the genetic integrity of populations and be of particular concern to endangered species. Hybridization between grey wolves and dogs has been documented in many wolf populations worldwide and is a prominent example of human‐mediated hybridization between a domesticated species and its wild relative. We analysed whole‐genome sequences from >200 wolves and >100 dogs to study admixture in Fennoscandian wolf populations. A principal component analysis of genetic variation and admixture showed that wolves and dogs were well‐separated, without evidence for introgression. Analyses of local ancestry revealed that wolves had <1% mixed ancestry, levels comparable to the degree of mixed ancestry in many dogs, and likely not resulting from recent wolf–dog hybridization. We also show that the founders of the Scandinavian wolf population were genetically inseparable from Finnish and Russian Karelian wolves, pointing at the geographical origin of contemporary Scandinavian wolves. Moreover, we found Scandinavian‐born animals among wolves sampled in Finland, demonstrating bidirectional gene flow between the Scandinavian Peninsula and eastern countries. The low incidence of admixture between wolves and dogs in Fennoscandia may be explained by the fact that feral dogs are rare in this part of Europe and that careful monitoring and management act to remove hybrids before they backcross into wolf populations.

## 1 INTRODUCTION

Hybridization between species or populations frequently occur in nature (Arnold, 1997; Harrison, 1993; Mallet, 2005) and may lead to introgression of genetic material between taxa. This can under certain conditions be advantageous for the recipient if introgressed variants increase fitness [“adaptive introgression” (e.g., Burgarella et al., 2019)], or even lead to the formation of new species [“hybrid speciation” (Mallet, 2007; Rieseberg, 1997; Schumer et al., 2014)].

For wild‐life management and conservation, however, hybridization is problematic (Allendorf et al., 2001; Jackiw et al., 2015). It threatens the genetic integrity of endangered populations and can lead to loss of genetic diversity and ultimately extinction (Rhymer & Simberloff, 1996; Wolf et al., 2001). A common strategy in monitored populations is to remove first‐generation hybrids, but there are rarely guidelines for how to handle backcrossed individuals, for example, concerning what levels of ancestry should be considered to classify an individual as *pure* or *admixed* (Allendorf et al., 2004; Jackiw et al., 2015). In practice, it has also been difficult to quantify levels of admixture. Rapidly developing whole‐genome sequencing technology is now changing the situation and dramatically increases the power of detecting even small amounts of admixture.

The incidence of hybridization is thought to have increased during the last centuries due to anthropogenic impact. Human‐mediated hybridization can for example occur through translocation of organisms or habitat modification (Allendorf et al., 2001). A particularly problematic type of hybridization is that between domesticated species and their wild relatives (Donfrancesco et al., 2019; Randi, 2008). This has been reported in a long list of taxa, including crops (Anderson & de Vicente, 2010), birds (Heikkinen et al., 2020; Lavretsky et al., 2019) and canids (Pilot et al., 2018), and is frowned upon due to the risk that artificially selected genes spread in the wild. The population sizes and ranges of domesticated species commonly outnumber that of wild ancestors. When domesticates are intentionally or unintentionally released, hybridization with wild populations can lead to loss of diversity and genetic swamping (reviewed in Todesco et al. (2016)).

The first species to be domesticated was the dog (*Canis lupus familiaris*), with an origin from grey wolves (*Canis lupus*) during Late Pleistocene. The exact time and location of the domestication event(s) are debated, but it has been suggested that dogs descend from a now extinct Eurasian wolf population and hence is a sister lineage to modern Eurasian wolves (reviewed in Freedman and Wayne (2017)). After the lineages split, there has been repeated admixture between dogs and wolves (e.g. Fan et al., 2016; Pilot et al., 2018; Sinding et al., 2020; Skoglund et al., 2015) resulting in bidirectional introgression. For example, an allele coding for black coat colour is thought to have been transferred from dogs to wolves (Anderson et al., 2009; Schweizer et al., 2018), and as an example of introgression in the opposite direction, adaptation to high‐altitude has been spread from Tibetan wolves to dogs in the same region (vonHoldt et al., 2017). Furthermore, wolves have a history of hybridization with coyotes in the New World (e.g. Wayne, 1993) and with jackals in southern Eurasia (Freedman et al., 2014), resulting in complicated admixture patterns.

More or less globally, the number of wolves has decreased substantially in the last hundreds of years due to hunting and expanding urbanization (e.g. Mech, 1995). In Europe, many wolf populations were driven to partial or complete extinction, but legal actions and conservation efforts have led to the return of wolves in many regions (Chapron et al., 2014; Kaczensky et al., 2013). However, many critical voices—especially from rural areas—are raised against increasing wolf populations, often with reference to the negative impact on livestock husbandry and game hunting (Hindrikson et al., 2017). The concern seems to be particularly strong in areas with recently refounded populations where wolves once were extirpated (Boitani, 2005; Dufresnes et al., 2019; Liberg, 2005). This is applicable to the Scandinavian population of grey wolves.

Wolves were once common over the entire Scandinavian Peninsula, but the population was driven to extinction in the late 1960s after extensive and long‐term prosecution. However, in the early 1980s, a new population was founded in southern Sweden by two single individuals (Wabakken et al., 2001). The fact that they appeared 1,000 km from the nearest wolf population in Finland and Russia rose suspicion that it was not a natural settlement. The population was closely monitored from early on (Wabakken et al., 2001), documenting the existence of one single wolf territory during the 1980s, where all wolves could be traced back to the two founders. A third founder appeared in 1991 and rescued the population from inbreeding depression (Vilà, Sundqvist et al., 2003). Since then, continuous expansion has led to a current population size of ≈450 individuals and about 70 territorial pairs (Wabakken et al., 2020). More recently, additional immigrants have reproduced (Åkesson et al., 2016; Åkesson & Svensson, 2020). As is the case for many other wolf populations around the world (Andersone et al., 2002; Godinho et al., 2011; Randi et al., 2014), possible hybridization with dogs (or even an origin including dogs) would have implications to management actions and conservation status of the population.

Here, we present a large‐scale admixture analysis based on whole‐genome sequences from >200 wolves from Scandinavia, Finland and Russia (Karelia), sampled over many years. We compare these data to a large number of dogs from multiple breeds and to wolves from other parts of the world. Our aim was to assess whether there is evidence for admixture between Fennoscandian (Scandinavia, Finland and Russian Karelia) wolves and dogs, and to address the genetic relationship among Fennoscandian wolf populations.

## 2 MATERIALS AND METHODS

### 2.1 Sequencing

We sequenced the genomes of 23 wolves from Finland and 15 from Russian Karelia to a mean coverage of 30×, following the same procedure as in Kardos et al. (2018) and Smeds et al. (2019). We also sequenced three F_1_ wolf–dog hybrids from southern Sweden that were identified during the yearly inventory and removed by protective hunting (Wabakken et al., 2018). In short, DNA was extracted from frozen tissue (Finnish and F_1_ hybrid samples) or dried pieces of skin (Russian samples) and sequencing libraries were prepared using the TruSeq Nano DNA Sample Preparation Kit, targeting an insert size of 350bp. Paired‐end sequencing with read length 150bp was performed on an Illumina HiSeq X Instrument.

### 2.2 Publicly available data

We used genome sequences from 98 Scandinavian and 75 Finnish wolves from Kardos et al. (2018) and Smeds et al. (2019). We also downloaded publicly available genome sequence data (Illumina paired‐end sequences) from 11 Chinese, three Russian and 10 (Arctic) North American wolves, as well as from 112 dogs. A list of all samples and their accession numbers is provided in Table S1. For dogs, we strived to include sequences from as many individuals as possible from Nordic and Arctic breeds, supplemented with a mix of breed dogs and “village” dogs. We grouped them into European (mostly modern breed dogs) and Asian dogs (breed dogs and village dogs), respectively, as previous studies have showed these groups to be genetically distinct (Frantz et al., 2016). We let Nordic–Arctic breeds form a separate group since it has been suggested that such breeds represent “ancient” or “basal” clades (Larson et al., 2012; vonHoldt et al., 2010). Moreover, most Nordic–Arctic breeds are morphologically more similar to wolves than other breeds, and might be more likely to interbreed with them.

### 2.3 Read mapping and variant detection

All reads were mapped onto the dog reference genome (CanFam3.1, (Lindblad‐Toh et al., 2005)) using BWA‐MEM version 0.7.17 (Li & Durbin, 2009), sorted with samtools version 1.9 (Li et al., 2009) and deduplicated with picard version 2.10.3 (http://broadinstitute.github.io/picard/) using default parameters. The newly sequenced samples were base‐recalibrated with GATK’s BaseRecalibrator v 3.8 (McKenna et al., 2010) following the “GATK Best Practices” (Van der Auwera et al., 2013). We used known polymorphic sites from Kardos et al. (2018) together with publicly available dog variation data from 219 individuals (accession number PRJEB25066, downloaded from https://www.ebi.ac.uk/eva/?eva‐study=PRJEB24066). The latter were included to account for variation in hybrids.

All samples were individually called for variants using GATK’s HaplotypeCaller v 3.8, and polymorphism data were then merged with GATK’s GenotypeGVCFs. We extracted biallelic single nucleotide polymorphisms (SNPs) and removed any sites with only heterozygous or homozygous calls. The data were filtered using GATKs VariantFiltration following Alternative protocol 2 in GATK Best Practices (VQSR was not performed due to the lack of a true reference wolf SNP set). We further removed sites with an overall average coverage below 10× (to reduce the incidence of sites with potentially missing alleles) or an average coverage above twice the genome‐wide coverage (to avoid problematic regions such as duplications, which might result in false SNPs), and removed any individual calls with a genotype quality less than 30. In the final set, we only kept sites with a minor allele count of ≥2 (corresponding to a minor allele frequency (MAF) of 0.003) and where less than 5% of the individuals had missing calls. All filtering steps were performed with vcftools version 0.1.15 (Danecek et al., 2011). The final VCF file was converted to plink format with vcftools. For all downstream analyses, only autosomal data (chromosome 1–38) were used.

Thinning of linked sites (referred to as “LD filtering”) for admixture and local ancestry analyses was performed with plink version 1.9 (Purcell et al., 2007) using the flag *‐‐indep‐pairwise 50kb 1 0.5* (meaning that for each pair of SNPs within 50‐kb windows with a stronger correlation coefficient than 0.5, one was removed). We also tested using a correlation threshold of 0.1, and this drastically reduced the number of markers, but did not affect the results.

### 2.4 Relatedness

As all Scandinavian‐born individuals are related to each other, we excluded them from analyses where unrelated samples were needed. Among immigrants to Scandinavia, two pairs of individuals were related at a level corresponding to that of full siblings; we excluded one individual from each of these pairs. For the Finnish and Russian populations, related samples were removed using plink *‐‐rel‐cutoff 0.05*. We also found high relatedness between a Finnish and a Russian individual, and between another Finnish individual and a Scandinavian immigrant. We removed the Finnish individual in both cases to even out the group sizes.

### 2.5 Principal component analysis

Principal components were calculated with plink version 2.0 using the flag *‐‐pca*. Per cent of variance explained was calculated from the.eigenval output. The first two PCs were used for plotting.

Inbreeding in Scandinavian wolves was obtained from Kardos et al. (2018), based on the proportion of the genome covered by runs of homozygosity (*F*_ROH_). The number of generations to the closest founding ancestor in this population was calculated from the Scandinavian pedigree (Åkesson & Svensson, 2016) using a perl script. All plots were drawn in R version 3.6.1 (R Core Team, 2019).

### 2.6 admixture analysis

admixture estimates the respective proportions of a given number of ancestries for an individual using a Bayesian approach (Alexander et al., 2009). Since the program assumes that individuals are unrelated, we only used an unrelated subset of immigrants, Finnish and Russian Karelian wolves as explained above, together with Chinese and Arctic North American wolves and all dogs. admixture analyses have been shown to be affected by unequal sample sizes (Meirmans, 2019; Puechmaille, 2016), and we did therefore not include available single wolf genomes from other parts of Europe (Spain, Portugal, Italy, Croatia, and Israel) and Asia (Iran and India).

We used admixture v1.3.0 with *K* = [2,10] and the *‐‐cv* flag to calculate cross‐validation errors for each *K*. We also assessed the best K using the method for uneven sample sizes suggested in Puechmaille (2016) and implemented in structureselector (Li & Liu, 2018). To further avoid sample size bias, we subsampled the set of European dogs as this was considerably larger than any other set, by randomly extracting 20 individuals.

### 2.7 Phasing variants

Filtered variants from all individuals were phased using shapeit4 (Delaneau et al., 2019) with N_e_ set to 1,500 and using recombination rates from the pedigree‐based genetic map developed for dog (Campbell et al., 2016).

### 2.8 Local ancestry analyses with pcadmix

pcadmix uses two pure populations as proxy for the ancestral populations and assigns each phased haplotype to any of the “ancestors” (Brisbin et al., 2012). We first used LD‐filtered SNPs from unrelated wolves and dogs (excluding individuals that had missing calls for more than 10% of the sites), as the two ancestral populations to assess admixture in the F_1_ hybrids, with the settings *‐ld 0* and various window sizes *‐w* ranging from 20 (default) to 200 SNPs in pcadmix version 3. As genetic position of markers is required, we used the above‐mentioned dog map from Campbell et al. (2016).

To assess admixture in Scandinavian wolves and immigrants to Scandinavia, we used unrelated Finnish, Russian Karelian, Chinese and North American wolves as the ancestral wolf population. For Finnish and Russian Karelian wolves, we chose ancestral wolf populations in a corresponding manner. In all three cases, all dogs (with <10% missing sites) were used as the ancestral dog population and we set *‐w* to 100.

To assess the robustness of the analysis, we performed multiple runs with different numbers of ancestral individuals and markers. When subsets of populations were used, the individuals were chosen randomly using the bash command *shuf*. For each size of the ancestral population, we repeated the analysis five times using different sets of individuals. To test the effect of varying number of markers, the marker set was reduced first by using a more stringent minor allele frequency threshold of either ≥0.05 or ≥0.10, and then by allowing only one marker for every 5, 10, 20, 30 and 50 kb, respectively, using the *‐‐thin* option in vcftools. For all pcadmix analyses, we calculated the dog ancestry as the sum of regions confidently assigned to dog from the forward–backward algorithm, that is only regions that met the default confidence threshold of 0.9.

To assess the fraction of wolf ancestry in dogs, we randomly extracted 50 dogs (and 50 wolves) with *shuf* to use as ancestral populations, while the remaining dogs were used as the admixed population for which ancestry was to be inferred. Wolf ancestry was calculated using the same method as for dog ancestry in wolves (see above).

### 2.9 Local ancestry analyses with elai

elai uses a two‐layer hidden Markov model to infer local ancestry (Guan, 2014). Just as pcadmix, it requires two ancestral populations as input, but it has the advantage that it does not require phased genomes. We ran elai with the same set‐up of individuals as for pcadmix, using the suggested settings *–s 30 –C 2 –c 10* (meaning 30 EM steps, two upper‐layer clusters and ten lower‐layer clusters), and two different numbers of admixing generations *(‐mg 10* or *–mg 100*, meaning number of generations during which admixture took place). We used the full set of SNPs (without LD filtering) as elai incorporates LD patterns in its model. Each set‐up was run five times, and the result was averaged over the runs.

## 3 RESULTS

### 3.1 Genetic clustering of individuals based on principal component analysis

After stringent filtering of whole‐genome sequences, we obtained a set of 3,456,384 autosomal SNPs from 210 wolves (97 Scandinavian, 98 Finnish and 15 Russian Karelian), 112 dogs and three wolf–dog F_1_ hybrids. Scandinavian wolves consisted of 85 animals born in Sweden or Norway, including the founder female sampled in 1985, and 12 immigrant wolves found mostly in northern Sweden and Norway. The F_1_ hybrids were three siblings from a litter born in the wild in southern Sweden 2017 as the result of a cross between a female wolf from the Scandinavian population and an unidentified dog (Wabakken et al., 2018).

**FIGURE 1:**
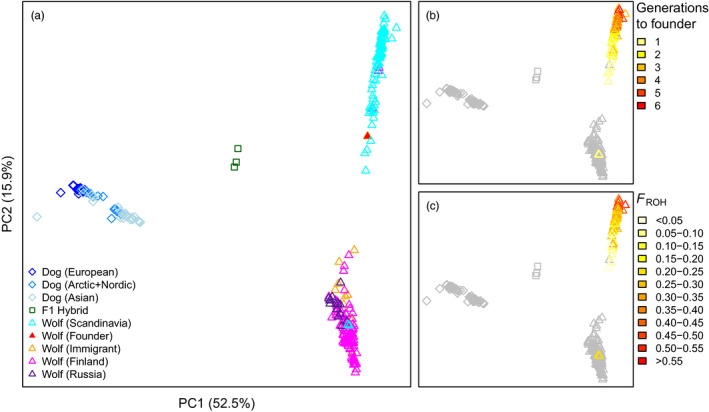
PCA of wolves and dogs based on whole‐genome sequence data. (a) All Scandinavian, Finnish and Russian Karelian wolves, three F_1_ hybrids and 112 downloaded dogs. (b–c) Same PCA as in 1a, but showing the effect of drift and inbreeding in Scandinavian wolves. The rest of the samples are shown in grey for better visibility. (b) Scandinavian‐born wolves coloured based on the number of generations to the closest founder. (c) Scandinavian‐born wolves coloured based on inbreeding estimated by F_ROH_

A PCA showed that dogs and wolves were well‐separated from each other, with the three F_1_ hybrids falling exactly in between the dog cluster and the Scandinavian wolf cluster (Figure 1a). Scandinavian‐born wolves separated from Finnish and Russian Karelian wolves along PC2, with the two latter groups being inseparable. The female founder of the Scandinavian population—related to practically all Scandinavian wolves—clustered with Scandinavian wolves, while all Scandinavian immigrants clustered with Finnish and Russian Karelian wolves.

The pattern described above came with some exceptions. The two Scandinavian‐born wolves seen in the Finnish–Russian Karelian cluster are direct offspring to an immigrant pair (translocated to southern Sweden), and thus not related to the rest of the Scandinavian population. Three individuals sampled in Finland clustered with Scandinavian wolves. Very high relatedness coefficients between each of these three and several Scandinavian‐born wolves suggest that they come from the Scandinavian population and have emigrated to Finland.

Although forming a distinct cluster, Scandinavian wolves were relatively spread over the PC2 axis. At first glance, it may seem surprising that individuals from a small and closed population do not group more tightly. In an attempt to explain this pattern, we considered the level of individual inbreeding (F_ROH_) and the number of generations to the closest founder (Figure 1b,c). It is clear that the most inbred samples cluster furthest away from Finnish and Russian Karelian samples. The distance also increased with the number of generations to the closest immigrant ancestor. This indicates that genetic drift has a large effect in the analysis of principal components of genetic variation.

**FIGURE 2:**
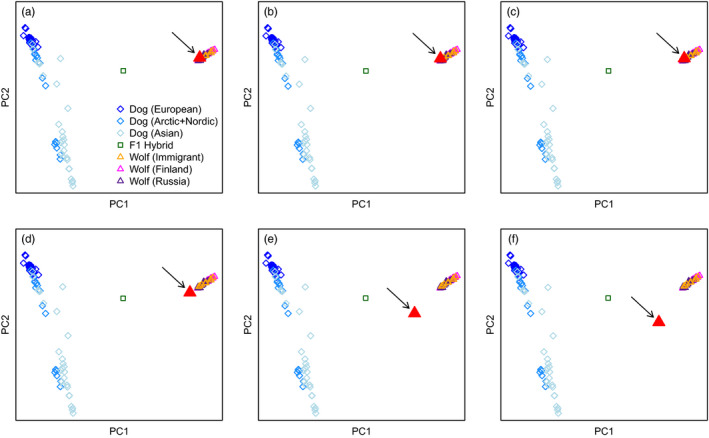
PCA using only unrelated wolves: nine immigrants, 15 Finnish, 13 Russian Karelian and one F_1_ hybrid. Each panel shows a specific test sample (large red triangle, marked with arrow). (a) Female founder of the Scandinavian wolf population. (b) Offspring to the first male founder. (c) Offspring to the second male founder. (d) Wolf from Bryansk (>1,000 km from Finland). (e) Wolf from Altai (>4,000 km from Finland). (f) Wolf from Chukotka (>6,000 km from Finland)

Since high relatedness among individuals may bias principal component analysis, we repeated the analysis including only unrelated samples (unrelated immigrants, unrelated Finnish and Russian Karelian individuals, one hybrid and dogs). To this set, we then added in turn individual test samples. The female founder of the Scandinavian population now clustered with Finnish–Russian Karelian wolves (Figure 2a). The two male founders of the Scandinavian population are not sampled, but direct offspring from both of them are. Testing in each case, one offspring from the male founders revealed that they also clustered with Finnish–Russian Karelian wolves (Figure 2b,c).

Clustering of founder/founder offspring together with Finnish–Russian Karelian wolves could potentially be due to attraction of any wolf samples to each other given their firm separation from dogs. To test this possibility, we performed the same analysis with three publicly available Russian wolf samples from localities at increasing distance to Finland (Bryansk, Altai, Chukotka). They all fell outside the cluster of Finnish–Russian Karelian wolves and further away so with increased geographical distance (Figure 2d–f). The clustering of Scandinavian and Finnish–Russian Karelian wolves therefore supports a Finnish–Russian origin of the Scandinavian wolf population.

### 3.2 Admixture analysis

**FIGURE 3:**
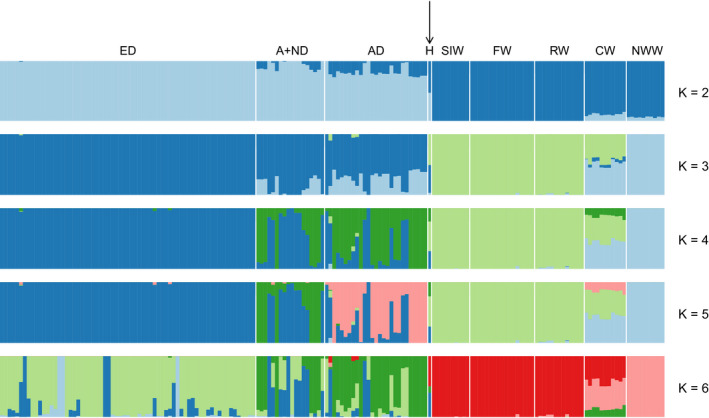
Result from admixture using *K* = 2–6 clusters. Cross‐validation errors give that *K* = 4 best explains the data. Abbreviations: ED—European dogs, A + ND—Arctic and Nordic dogs, AD—Asian dogs, H—F_1_ wolf–dog hybrid, SIW—Scandinavian immigrant wolves, FW—Finnish wolves, RW—Russian Karelian wolves, CW—Chinese wolves, NWW—New World wolves. The F_1_ hybrid is marked with an arrow for visibility

To more broadly investigate the genetic structure of wolf populations, we used the program admixture and also included publicly available wolf genome sequences from China and Arctic North America. In addition to the original filtering, we also filtered for linkage disequilibrium to avoid dependency between markers, leaving 1,370,987 SNP markers. The data were best explained by four clusters using cross‐validation errors (*K* = 4, see Figure S1), with European dogs as one distinct group, wolves from Scandinavia, Finland and Russian Karelia as another, and wolves from North America as a third. The fourth component was associated with several groups (Figure 3). Dogs from Nordic–Arctic and Asian breeds shared ancestry with other dogs and the fourth component. Finally, Chinese wolves appeared to have a more mixed ancestry, mainly shared with the two other wolf groups but also including the fourth component. A few wolves from Russian Karelia, and one of the Finnish individuals, had small fractions of ancestry shared with Chinese and New World wolves (Figure 3, Figure S2). When *K* was instead optimized using the methods suggested by Puechmaille (2016), five clusters best explained the data. This added a component in Asian dogs and Chinese wolves, but did not impact the ancestry of Fennoscandian wolves.

The F_1_ hybrid shared half its ancestry with wolves from Scandinavia, Finland and Russian Karelia, and half with dogs, as expected. Its “dog ancestry” was more similar to Nordic–Arctic and Asian dogs than to European breed dogs. Since the group of European dogs was substantially larger than any other group, we also performed the analyses with this group down‐sampled to the same size as the others, but this did not affect the results (Figure S3).

### 3.3 Local ancestry

Neither PCA nor admixture indicated genome‐wide admixture between Fennoscandian wolves and dogs. This does not exclude that there are smaller genomic segments with a different ancestry in wolf populations, potentially reflecting introgression sometime in the past. Current methods for the analysis of local ancestry patterns along the chromosomes require either phased genomes, known “pure” ancestral populations, or knowledge about the number of generations since admixture or number of admixed individuals. Each of these aspects has its own limitations (see Discussion).

**FIGURE 4:**
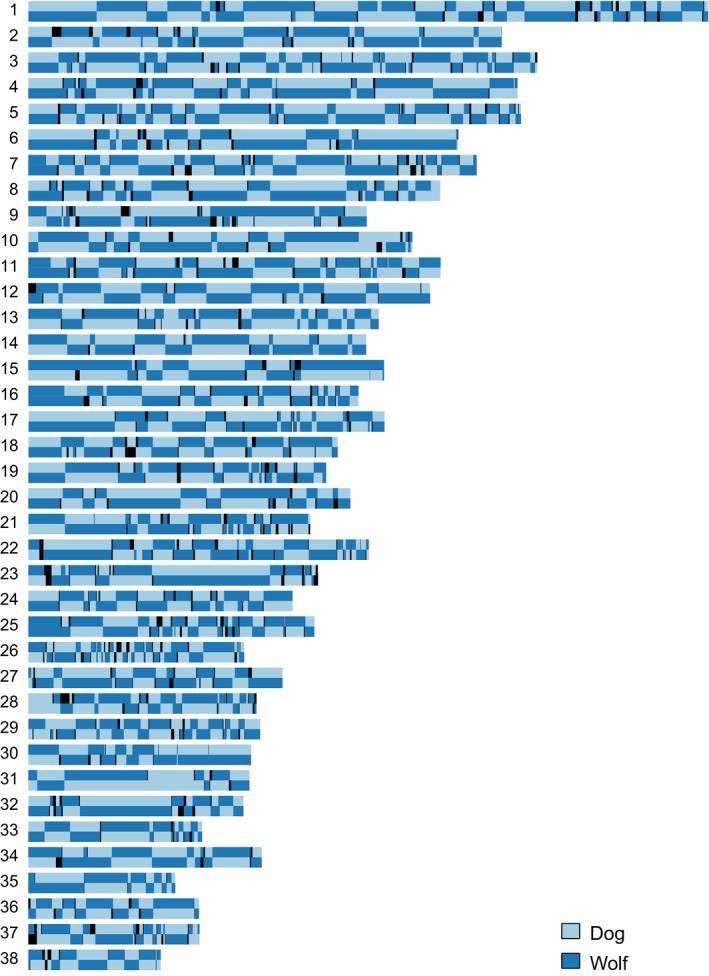
Results from pcadmix for one F_1_ wolf–dog hybrid using window size = 100 SNPs. The chromosome pairs are drawn on top of each other. Light blue colour—assigned dog ancestry and dark blue—assigned wolf ancestry. Black colour indicates uncertain ancestry assignment. With perfectly phased chromosomes, one chromosome of each pair should be dark blue and one light blue

In the absence of chromosomes with known phasing, we used shapeit4 to statistically phase all samples. We then applied pcadmix to the known F_1_ hybrids, including all dogs and unrelated wolves as “pure” ancestors, to assess how well the phasing performed. As the F_1_ hybrids have one dog chromosome and one wolf chromosome for every chromosome pair, any disruption in this pattern must be caused by a switch error in the statistical phasing. As shown in Figure 4, the “mosaic” appearance of each chromosome pair in F_1_ hybrids means that statistical phasing suffered from multiple switch errors per chromosome (0.218–0.234 switches/Mb using a window size of 100 SNPs, see Table S2 for different window sizes). There were also genomic regions with only dog, or only wolf, ancestry inferred on both chromosome copies (3.9%–5.1% for the three hybrids).

With the phasing errors in mind, we also ran pcadmix on Scandinavian, Finnish and Russian Karelian wolves, respectively, to assess whether there were genomic regions in individual wolves that could potentially be of dog ancestry; all other unrelated wolves were used as one of the ancestral populations. This suggested that 0.09%–1.08% of the genome of Scandinavian wolves had mixed ancestry (mean = 0.48%). The proportions for Finnish and Russian Karelian wolves were similar; see Table 1. As the Scandinavian samples were collected over a 30‐year time period, we divided them into four temporal groups and compared the dog ancestry between groups. We found no significant difference in the proportion of dog ancestry among the groups (Figure S4).

**TABLE 1:** Proportion of the genome with mixed ancestry estimated with pcadmix and elai (10 or 100 mixing generations)

**Population**	**Mixed ancestry pcadmix**	**Mixed ancestry elai (mg = 10)**	**Mixed ancestry elai (mg = 100)**
Scandinavian^a^	0.0009–0.0108 (mean 0.0048)	0.0011–0.0113 (mean 0.0060)	0.0022–0.0123 (mean 0.0076)
Finnish	0.0003–0.0145 (mean 0.0037)	0.0010–0.0168 (mean 0.0057)	0.0028–0.0190 (mean 0.0083)
Russian Karelian	0.0014–0.0132 (mean 0.0047)	0.0019–0.0155 (mean 0.0071)	0.0045–0.0193 (mean 0.0105)
F_1_ Hybrids	0.4696–0.4716 (mean 0.4707)	0.4995–0.5020 (mean 0.5005)	0.4937–0.4960 (mean 0.4945)

^a^ Excluding hybrids but including immigrants.

As a complement, we assessed the level of wolf ancestry in dogs using multiple random subsets as the ancestral dog population. The assigned wolf ancestry differed significantly between different groups of dogs (Figure S5). Asian dogs showed the highest levels of wolf ancestry (mean = 1.1%), while European dogs had the lowest (mean = 0.1%). Nordic–Arctic breeds had a mean wolf ancestry of 0.52%.

**FIGURE 5:**
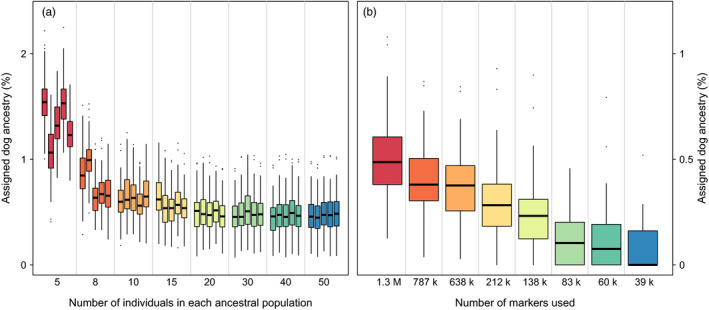
Assigned dog ancestry in the Scandinavian population using different numbers of individuals and markers. (a) Five sets of wolves and dogs were chosen randomly as ancestral population for each size. (b) Subsampling of markers evenly spread along the genome

We repeated the pcadmix analysis with different numbers and combinations of ancestral individuals, and also down‐sampled the number of markers. The extent of assigned dog ancestry changed considerably among different runs. It increased when using very few ancestral individuals, and decreased with the number of markers used (Figure 5). Over all runs, the mixed ancestry was never larger than 2.25% for a single individual.

We also assessed the amount of dog ancestry using the software elai that does not require phased genomes, but needs information on the number of mixed generations. Assuming 10 mixing generations, the amount of inferred dog ancestry was in a similar range as for pcadmix. Assuming 100 mixing generations increased the numbers slightly (Table 1).

Both pcadmix and elai indicated that the blocks of potential dog ancestry were unevenly spread across the genome. For most individuals, the blocks were concentrated to a handful of chromosomes, while the remaining chromosomes showed no dog ancestry at all. The length of each individual block is not meaningful due to the above‐mentioned problem with switch errors, but the distribution of blocks over the genomes is shown in Figure S6 for each population separately. We especially note multiple regions on chromosome 31 that were present in both Scandinavian, Finnish and Russian Karelian wolves. The Scandinavian population showed fewer regions in total, but many regions reoccurred in multiple individuals, consistent with the high relatedness between samples. Almost all regions seen in the Scandinavian population were seen also in either the Finnish or the Russian Karelian population in at least one individual.

## 4 DISCUSSION

The relationship between wolves and humans is complex, and deeply rooted in human history. Notably, people's attitude to wolves changed by the transition from hunter gathering to farming. Wolves later became symbols of evilness in religion and culture, including in fairy tales. Now, the grey wolf is often seen as a flagship species for conservation and the handling of conflicts between humans and endangered species. Wolf–dog hybridization is a key issue worldwide since it has strong implications to the management of wolf populations. For example, several recent studies have addressed how it may affect policy making (Donfrancesco et al., 2019; Salvatori et al., 2020) and how thresholds for identification of admixed individuals should be set (Caniglia et al., 2020).

Wolf–dog hybridization has been documented twice in the contemporary Scandinavian wolf population: in Norway 1999 (Vilà, Walker et al., 2003) and in Sweden 2017 (Wabakken et al., 2018). In both cases, a female wolf mated with an unknown male dog. Management aimed to remove all F_1_ offspring, which is thought to have been successful. Although the population is subject to detailed genetic monitoring (Liberg et al., 2012; Wabakken et al., 2020), where an exceptionally large proportion of the population is represented by a DNA sample (Bischof et al., 2019), we cannot formally exclude that there have been other hybridization events. Moreover, the number of markers used in monitoring programmes is typically not sufficient for detecting slightly admixed individuals. In Finland, three cases of wolf–dog hybridization also led to immediate culling of F_1_ offspring (Ministry of Agriculture & Forestry, 2019).

### 4.1 No evidence for wolf–dog hybridization threatening the genetic integrity of Fennoscandian wolf populations

Both the PCA and admixture analyses clearly showed that neither Scandinavian nor Finnish or Russian Karelian wolves have detectable levels of dog ancestry. From this, we conclude that potential hybridization between wolves and dogs has not left a significant imprint on the genetic composition of wolf populations in northernmost Europe. The inclusion of known F_1_ hybrids in the analyses served as excellent control samples to reach this conclusion. Their positioning exactly between Scandinavian wolves and dogs in the PCA, and the precise 50/50 proportions of assignment to Scandinavian wolves and dogs in admixture, validates the approaches taken.

In the absence of genome‐wide evidence for wolf–dog hybridization in the studied populations, we attempted to elucidate whether there are specific genomic regions that bear signature of admixture. After statistical phasing of sequenced genomes, a number of regions with mixed ancestry were identified using pcadmix. Taken together, these regions corresponded to <1% of individual genomes, and the proportions were similar for Scandinavian, Finnish and Russian Karelian wolves; most regions were also shared among these populations. The proportion did not change over time in the Scandinavian population. These observations suggest that the signatures of local mixed ancestry in wolf genomes cannot be taken as an indication of a low frequency of relatively recent wolf–dog hybridization. This is further supported by the fact that we found similar levels of genomic regions with mixed ancestry when performing the reciprocal analysis of different groups of dogs (i.e. testing for signatures of wolf ancestry in dog genomes), including in carefully managed breed dogs. The common pattern seen in wolf and dog genomes could indicate that technical issues underlie signatures of mixed ancestry, which we elaborate on below.

### 4.2 Methodological aspects

The statistical phasing was evidently associated with switch errors, on average one every 4–5 Mb. This could be concluded from the analysis of known F_1_ hybrids, which again thus proved to be very valuable to our study. It is unclear whether incorrect phasing will impact on the identification of local regions of mixed ancestry, but it will clearly affect the resulting block lengths. A number of previous studies have used haplotype block length to infer timing of admixture events; shorter block will require more recombination events and hence more generations since admixture to be formed (see, e.g., Galaverni et al., 2017; Gómez‐Sánchez et al., 2018). This approach will obviously be biased if haplotype blocks are incorrectly inferred, as we showed was the case for F_1_ hybrids. Switch errors will result in underestimation of ancestral block lengths and, accordingly, overestimation of the number of generations since admixture.

Also, the close genetic relationship between wolves and dogs—recall that dogs were domesticated from wolves only 10,000–30,000 years ago (Fan et al., 2016)—can potentially be problematic for detecting low levels of introgression arising from wolf–dog hybridization. Specifically, signatures of mixed ancestry may represent shared ancestral variation rather than hybridization. Extended periods of gene flow between wolves and prehistorical dogs during the initial phase of domestication could have counteracted the process of lineage sorting.

Phasing is not the only methodological aspect that can bias estimates of the incidence of wolf–dog hybridization and dog introgression. Another key issue is the identification of appropriate reference (ancestral) populations, which is needed in software such as pcadmix and elai, and also important when setting thresholds for defining admixed individuals (see Caniglia et al., 2020). To say a priori if an individual sampled in the wild is genetically pure might be difficult, if not impossible, so often admixture or structure (Pritchard et al., 2000) is used to identify seemingly pure individuals to be included in a reference population. But with increasing number of markers, the power to detect even small introgressed regions, and older admixture, also increases (McFarlane & Pemberton, 2019). In a system with occasional/rare admixture over a long period of time, most individuals will have at least some degree of admixture, and even minuscule fractions of introgressed blocks can be detected if a sufficiently large number of markers are used (like in analyses of whole‐genome sequences). As a consequence, a pure reference population might be impossible to obtain.

The number of individuals used in the reference populations clearly affected the inference of the degree of dog ancestry in wolf populations (see Figure 5). Using less than 10 individuals per population (as was done by Gómez‐Sánchez et al. (2018) for a wolf reference population) increased the assigned dog ancestry more than twofold, compared with using 20 or more individuals. The levels of mixed ancestry were stabilized with higher number of individuals. When subsampling the number of markers, the assigned dog ancestry decreased; with less than 50,000 markers, the mean mixed ancestry approached zero. On the other hand, using more markers than we had access to in our filtered data set might increase the level of assigned dog ancestry (as discussed above and seen from the trend in Figure 5b), when the resolution to detect smaller regions increases.

Local ancestry assignment methods are with few exceptions developed for human data, where large reference panels are available in terms of both unrelated individuals and number of loci. There are methods for analyses of local ancestry that do not require fixed “ancestral” populations, such as LAMP (Sankararaman et al., 2008) and MOSAIC (Salter‐Townshend & Myers, 2019). However, they require a large number of unrelated samples for each population and are developed to distinguish between very closely related populations. We acknowledge that our data sets are not sufficiently large for this type of analysis.

### 4.3 The incidence of wolf–dog hybridization in different European wolf populations

Wolf–dog hybridization has been recorded in all investigated extant wolf populations in Europe (Salvatori et al., 2020). F_2_ and F_3_ backcrosses have been found in Italy, Spain, Russia, Ukraine, Belarus and Israel (Galaverni et al., 2017; Gómez‐Sánchez et al., 2018; Pilot et al., 2018, 2019) and in many countries a large number of wolves with admixture levels of 1%–8% have been identified, indicative of further backcrossing. The levels of admixture thus seem to be lower in Fennoscandian wolves compared with other European populations. This might be explained by the fact that feral dogs are less common in Fennoscandia than in other parts of Europe, reducing the number of contacts between wolves and dogs. Moreover, the Scandinavian wolf population is closely monitored and carefully managed (Liberg et al., 2012). The two known cases of wolf–dog hybridization in Sweden and Norway and the three cases in Finland immediately led to culling of hybrid offspring. Since wolf–dog hybridization might be hard to prevent in most countries, a general strategy for maintaining genetic integrity of wolf populations is indeed to avoid backcrossing of hybrid offspring.

### 4.4 Population structure of Fennoscandian wolves

The separation of Scandinavian from Finnish–Russian Karelian wolves in a PCA including all samples came as a surprise at first, considering that the Scandinavian population supposedly was founded by immigrants from Finland and/or Russia (Wabakken et al., 2001). However, the separation was clearly the result of a few generations of drift and inbreeding in the Scandinavian population, with the most inbred individuals also most separated from Finnish–Russian Karelian wolves. When using only unrelated individuals, Scandinavian and Finnish–Russian Karelian wolves clustered. Close within‐population relatedness can confound principal component analysis such that populations look more distinct than they (in some sense) are. In extreme cases, the resulting pattern might reflect family differences rather than population differences. This highlights the importance of sample awareness, especially when studying small inbred populations where it might be difficult, if not impossible, to obtain unrelated samples.

Importantly, in the analysis of unrelated individuals, the clustering of the female founder, and offspring to the two (unsampled) male founders, with Finnish–Russian Karelian wolves clearly points at a Finnish–Russian origin of the Scandinavian wolf population. It also shows that neither of the first three founders had dog ancestry. It should be pointed out that, in theory, a single individual from a different population might cluster with another population, seemingly indicating the latter to represent the source population, just because of lack of more related individuals to cluster with. However, in our case, the fact that three other Russian wolves sampled further away from Finland did not cluster with Finnish–Russian Karelian wolves, provides indirect support to the conclusion that the clustering of Scandinavian founders with Finnish–Russian Karelian reflects the origin of the Scandinavian population.

Ever since the re‐establishment of the Scandinavian wolf population in the early 1980s, there has been a more or less regular influx of immigrants to northern Sweden and Norway from the east (Åkesson et al., 2016; Seddon et al., 2006). A Finnish–Russian origin of immigrants is confirmed by our analyses. Our study also demonstrates dispersal in the opposite direction: three Scandinavian‐born individuals were sampled in Finland. There are no obvious reasons to believe that immigration and emigration of wolves to/from Scandinavia are only a recent phenomenon. In contrast, when wolves were once common throughout Europe, including the whole Scandinavian peninsula (in Sweden, more than 500 wolves were annually killed in the early 19th century (Flagstad et al., 2003)), a continuous distribution range connecting Scandinavia with Finland and Russia must have been associated with ample gene flow. Therefore, and given large population sizes (counteracting genetic drift), Scandinavian wolves may not have been genetically differentiated from Finnish and Russian wolves, or at least not more than expected from isolation by distance. Such a scenario has bearing on conservation aspects such as the genetic uniqueness of Scandinavian wolves and the possibility that remnants of a historical population in Scandinavia contributed to the founding of the contemporary population. If one or more of the three founders was actually not an immigrant but represented an animal left from the historical population (supposedly extinct in the 1960s–1970s), it may still have been part of the same gene pool as immigrant wolves.

## Supporting information

Supplementary Material

## Data Availability

Raw sequence reads are submitted to ENA, accession number PRJEB39198. The filtered variant file (VCF) is available at the Dryad Digital Repository https://doi.org/10.5061/dryad.8gtht76n6, and bash commands and perl scripts are available on Github: https://github.com/linneas/fennoscandian_wolf.
