# Supplementary material for: Whole‐genome analyses provide no evidence for dog introgression in Fennoscandian wolf populations
Source: Evol Appl. 2020 Nov 9;14(3):721–34. doi: 10.1111/eva.13151 (PMC7980305; doi:10.1111/eva.13151)
Supplement: Supplementary file 1 — Supplementary Material [file EVA-14-721-s001.pdf]

Supplementary Materials

Supplementary tables

Table S1. List of all samples used in the study.

Name	Type	Sample location (wolves) Breed (dogs)	Sample accession	Study accession	Citation
D-00-12	Grey wolf	Scandinavia	ERS1754868	PRJEB20635	Kardos et al. 2018
D-00-15	Grey wolf	Scandinavia	ERS1754869	PRJEB20635	Kardos et al. 2018
D-01-18	Grey wolf	Scandinavia	ERS1754870	PRJEB20635	Kardos et al. 2018
D-05-18	Grey wolf	Scandinavia	ERS1754871	PRJEB20635	Kardos et al. 2018
D-05-23	Grey wolf	Scandinavia	ERS1754872	PRJEB20635	Kardos et al. 2018
D-06-14	Grey wolf	Scandinavia	ERS1754873	PRJEB20635	Kardos et al. 2018
D-06-16	Grey wolf	Scandinavia	ERS1754874	PRJEB20635	Kardos et al. 2018
D-07-09	Grey wolf	Scandinavia	ERS1754875	PRJEB20635	Kardos et al. 2018
D-07-16	Grey wolf	Scandinavia	ERS1754876	PRJEB20635	Kardos et al. 2018
D-07-17	Grey wolf	Scandinavia	ERS1754877	PRJEB20635	Kardos et al. 2018
D-07-24	Grey wolf	Scandinavia	ERS1754878	PRJEB20635	Kardos et al. 2018
D-07-28	Grey wolf	Scandinavia	ERS1754879	PRJEB20635	Kardos et al. 2018
D-08-08	Grey wolf	Scandinavia	ERS1754880	PRJEB20635	Kardos et al. 2018
D-08-10	Grey wolf	Scandinavia	ERS1754881	PRJEB20635	Kardos et al. 2018
D-08-19	Grey wolf	Scandinavia	ERS1754882	PRJEB20635	Kardos et al. 2018
D-08-20	Grey wolf	Scandinavia	ERS1754883	PRJEB20635	Kardos et al. 2018
D-08-21	Grey wolf	Scandinavia	ERS1754884	PRJEB20635	Kardos et al. 2018
D-10-15	Grey wolf	Scandinavia	ERS1754885	PRJEB20635	Kardos et al. 2018
D-10-20	Grey wolf	Scandinavia	ERS1754886	PRJEB20635	Kardos et al. 2018
D-10-23	Grey wolf	Scandinavia	ERS1754887	PRJEB20635	Kardos et al. 2018
D-10-29	Grey wolf	Scandinavia	ERS1754888	PRJEB20635	Kardos et al. 2018
D-10-30	Grey wolf	Scandinavia	ERS1754889	PRJEB20635	Kardos et al. 2018
D-10-44	Grey wolf	Scandinavia	ERS1754890	PRJEB20635	Kardos et al. 2018
D-10-50	Grey wolf	Scandinavia	ERS1754891	PRJEB20635	Kardos et al. 2018
D-10-53	Grey wolf	Scandinavia	ERS1754892	PRJEB20635	Kardos et al. 2018
D-10-68	Grey wolf	Scandinavia	ERS1754893	PRJEB20635	Kardos et al. 2018
D-11-17	Grey wolf	Scandinavia	ERS1754894	PRJEB20635	Kardos et al. 2018
D-11-22	Grey wolf	Scandinavia	ERS1754895	PRJEB20635	Kardos et al. 2018
D-11-58	Grey wolf	Scandinavia	ERS1754896	PRJEB20635	Kardos et al. 2018
D-77-01	Grey wolf	Scandinavia	ERS1754897	PRJEB20635	Kardos et al. 2018
D-79-01	Grey wolf	Scandinavia	ERS1754898	PRJEB20635	Kardos et al. 2018
D-84-03	Grey wolf	Scandinavia	ERS1754899	PRJEB20635	Kardos et al. 2018
D-85-01	Grey wolf	Scandinavia	ERS1754900	PRJEB20635	Kardos et al. 2018
D-85-02	Grey wolf	Scandinavia	ERS1754901	PRJEB20635	Kardos et al. 2018
D-86-01	Grey wolf	Scandinavia	ERS1754902	PRJEB20635	Kardos et al. 2018
D-89-01	Grey wolf	Scandinavia	ERS1754903	PRJEB20635	Kardos et al. 2018
D-89-03	Grey wolf	Scandinavia	ERS1754904	PRJEB20635	Kardos et al. 2018
D-91-01	Grey wolf	Scandinavia	ERS1754905	PRJEB20635	Kardos et al. 2018
D-92-01	Grey wolf	Scandinavia	ERS1754906	PRJEB20635	Kardos et al. 2018
D-92-02	Grey wolf	Scandinavia	ERS1754907	PRJEB20635	Kardos et al. 2018
D-92-05	Grey wolf	Scandinavia	ERS1754908	PRJEB20635	Kardos et al. 2018
D-92-06	Grey wolf	Scandinavia	ERS1754909	PRJEB20635	Kardos et al. 2018

D-93-01	Grey wolf	Scandinavia	ERS1754910	PRJEB20635	Kardos et al. 2018
D-93-02	Grey wolf	Scandinavia	ERS1754911	PRJEB20635	Kardos et al. 2018
D-93-03	Grey wolf	Scandinavia	ERS1754912	PRJEB20635	Kardos et al. 2018
D-94-01	Grey wolf	Scandinavia	ERS1754913	PRJEB20635	Kardos et al. 2018
D-96-01	Grey wolf	Scandinavia	ERS1754914	PRJEB20635	Kardos et al. 2018
D-99-02	Grey wolf	Scandinavia	ERS1754915	PRJEB20635	Kardos et al. 2018
G100-12	Grey wolf	Scandinavia	ERS1754916	PRJEB20635	Kardos et al. 2018
G100-14	Grey wolf	Scandinavia	ERS1754917	PRJEB20635	Kardos et al. 2018
G106-13	Grey wolf	Scandinavia	ERS1754918	PRJEB20635	Kardos et al. 2018
G109-11	Grey wolf	Scandinavia	ERS1754919	PRJEB20635	Kardos et al. 2018
G110-11	Grey wolf	Scandinavia	ERS1754920	PRJEB20635	Kardos et al. 2018
G111-14	Grey wolf	Scandinavia	ERS1754921	PRJEB20635	Kardos et al. 2018
G126-13	Grey wolf	Scandinavia	ERS1754922	PRJEB20635	Kardos et al. 2018
G139-12	Grey wolf	Scandinavia	ERS1754923	PRJEB20635	Kardos et al. 2018
G174-13	Grey wolf	Scandinavia	ERS1754924	PRJEB20635	Kardos et al. 2018
G175-13	Grey wolf	Scandinavia	ERS1754925	PRJEB20635	Kardos et al. 2018
G23-13	Grey wolf	Scandinavia	ERS1754926	PRJEB20635	Kardos et al. 2018
G24-14	Grey wolf	Scandinavia	ERS1754927	PRJEB20635	Kardos et al. 2018
G31-13	Grey wolf	Scandinavia	ERS1754928	PRJEB20635	Kardos et al. 2018
G32-12	Grey wolf	Scandinavia	ERS1754929	PRJEB20635	Kardos et al. 2018
G32-15	Grey wolf	Scandinavia	ERS1754930	PRJEB20635	Kardos et al. 2018
G34-10	Grey wolf	Scandinavia	ERS1754931	PRJEB20635	Kardos et al. 2018
G37-10	Grey wolf	Scandinavia	ERS1754932	PRJEB20635	Kardos et al. 2018
G47-11	Grey wolf	Scandinavia	ERS1754933	PRJEB20635	Kardos et al. 2018
G50-12	Grey wolf	Scandinavia	ERS1754934	PRJEB20635	Kardos et al. 2018
G58-15	Grey wolf	Scandinavia	ERS1754935	PRJEB20635	Kardos et al. 2018
G67-15	Grey wolf	Scandinavia	ERS1754936	PRJEB20635	Kardos et al. 2018
G82-10	Grey wolf	Scandinavia	ERS1754937	PRJEB20635	Kardos et al. 2018
G87-12	Grey wolf	Scandinavia	ERS1754938	PRJEB20635	Kardos et al. 2018
G9-05	Grey wolf	Scandinavia	ERS1754939	PRJEB20635	Kardos et al. 2018
G97-13	Grey wolf	Scandinavia	ERS1754940	PRJEB20635	Kardos et al. 2018
M-00-09	Grey wolf	Scandinavia	ERS1754941	PRJEB20635	Kardos et al. 2018
M-00-10	Grey wolf	Scandinavia	ERS1754942	PRJEB20635	Kardos et al. 2018
M-01-04	Grey wolf	Scandinavia	ERS1754943	PRJEB20635	Kardos et al. 2018
M-01-06	Grey wolf	Scandinavia	ERS1754944	PRJEB20635	Kardos et al. 2018
M-01-10	Grey wolf	Scandinavia	ERS1754945	PRJEB20635	Kardos et al. 2018
M-02-15	Grey wolf	Scandinavia	ERS1754946	PRJEB20635	Kardos et al. 2018
M-03-06	Grey wolf	Scandinavia	ERS1754947	PRJEB20635	Kardos et al. 2018
M-03-07	Grey wolf	Scandinavia	ERS1754948	PRJEB20635	Kardos et al. 2018
M-05-01	Grey wolf	Scandinavia	ERS1754949	PRJEB20635	Kardos et al. 2018
M-05-07	Grey wolf	Scandinavia	ERS1754950	PRJEB20635	Kardos et al. 2018
M-06-03	Grey wolf	Scandinavia	ERS1754951	PRJEB20635	Kardos et al. 2018
M-06-04	Grey wolf	Scandinavia	ERS1754952	PRJEB20635	Kardos et al. 2018
M-07-02	Grey wolf	Scandinavia	ERS1754953	PRJEB20635	Kardos et al. 2018
M-07-06	Grey wolf	Scandinavia	ERS1754954	PRJEB20635	Kardos et al. 2018
M-09-03	Grey wolf	Scandinavia	ERS1754955	PRJEB20635	Kardos et al. 2018
M-09-05	Grey wolf	Scandinavia	ERS1754956	PRJEB20635	Kardos et al. 2018
M-09-17	Grey wolf	Scandinavia	ERS1754957	PRJEB20635	Kardos et al. 2018
M-10-04	Grey wolf	Scandinavia	ERS1754958	PRJEB20635	Kardos et al. 2018
M-10-10	Grey wolf	Scandinavia	ERS1754959	PRJEB20635	Kardos et al. 2018
M-11-02	Grey wolf	Scandinavia	ERS1754960	PRJEB20635	Kardos et al. 2018

M-98-01	Grey wolf	Scandinavia	ERS1754961	PRJEB20635	Kardos et al. 2018
M-98-02	Grey wolf	Scandinavia	ERS1754962	PRJEB20635	Kardos et al. 2018
M-98-03	Grey wolf	Scandinavia	ERS1754963	PRJEB20635	Kardos et al. 2018
M-98-08	Grey wolf	Scandinavia	ERS1754964	PRJEB20635	Kardos et al. 2018
W10	Grey wolf	Finland	ERS2672862	PRJEB28342	Smeds et al. 2019
W100	Grey wolf	Finland	ERS2672863	PRJEB28342	Smeds et al. 2019
W101	Grey wolf	Finland	ERS2672864	PRJEB28342	Smeds et al. 2019
W11	Grey wolf	Finland	ERS2672865	PRJEB28342	Smeds et al. 2019
W12	Grey wolf	Finland	ERS2672866	PRJEB28342	Smeds et al. 2019
W13	Grey wolf	Finland	ERS2672867	PRJEB28342	Smeds et al. 2019
W14	Grey wolf	Finland	ERS2672868	PRJEB28342	Smeds et al. 2019
W15	Grey wolf	Finland	ERS2672869	PRJEB28342	Smeds et al. 2019
W17	Grey wolf	Finland	ERS2672870	PRJEB28342	Smeds et al. 2019
W19	Grey wolf	Finland	ERS2672871	PRJEB28342	Smeds et al. 2019
W20	Grey wolf	Finland	ERS2672872	PRJEB28342	Smeds et al. 2019
W21	Grey wolf	Finland	ERS2672873	PRJEB28342	Smeds et al. 2019
W22	Grey wolf	Finland	ERS2672874	PRJEB28342	Smeds et al. 2019
W24	Grey wolf	Finland	ERS2672875	PRJEB28342	Smeds et al. 2019
W25	Grey wolf	Finland	ERS2672876	PRJEB28342	Smeds et al. 2019
W26	Grey wolf	Finland	ERS2672877	PRJEB28342	Smeds et al. 2019
W27	Grey wolf	Finland	ERS2672878	PRJEB28342	Smeds et al. 2019
W31	Grey wolf	Finland	ERS2672879	PRJEB28342	Smeds et al. 2019
W34	Grey wolf	Finland	ERS2672880	PRJEB28342	Smeds et al. 2019
W35	Grey wolf	Finland	ERS2672881	PRJEB28342	Smeds et al. 2019
W36	Grey wolf	Finland	ERS2672882	PRJEB28342	Smeds et al. 2019
W38	Grey wolf	Finland	ERS2672883	PRJEB28342	Smeds et al. 2019
W41	Grey wolf	Finland	ERS2672884	PRJEB28342	Smeds et al. 2019
W44	Grey wolf	Finland	ERS2672885	PRJEB28342	Smeds et al. 2019
W46	Grey wolf	Finland	ERS2672886	PRJEB28342	Smeds et al. 2019
W5	Grey wolf	Finland	ERS2672887	PRJEB28342	Smeds et al. 2019
W50	Grey wolf	Finland	ERS2672888	PRJEB28342	Smeds et al. 2019
W51	Grey wolf	Finland	ERS2672889	PRJEB28342	Smeds et al. 2019
W52	Grey wolf	Finland	ERS2672890	PRJEB28342	Smeds et al. 2019
W53	Grey wolf	Finland	ERS2672891	PRJEB28342	Smeds et al. 2019
W57	Grey wolf	Finland	ERS2672892	PRJEB28342	Smeds et al. 2019
W58	Grey wolf	Finland	ERS2672893	PRJEB28342	Smeds et al. 2019
W59	Grey wolf	Finland	ERS2672894	PRJEB28342	Smeds et al. 2019
W6	Grey wolf	Finland	ERS2672895	PRJEB28342	Smeds et al. 2019
W60	Grey wolf	Finland	ERS2672896	PRJEB28342	Smeds et al. 2019
W63	Grey wolf	Finland	ERS2672897	PRJEB28342	Smeds et al. 2019
W64	Grey wolf	Finland	ERS2672898	PRJEB28342	Smeds et al. 2019
W65	Grey wolf	Finland	ERS2672899	PRJEB28342	Smeds et al. 2019
W66	Grey wolf	Finland	ERS2672900	PRJEB28342	Smeds et al. 2019
W67	Grey wolf	Finland	ERS2672901	PRJEB28342	Smeds et al. 2019
W68	Grey wolf	Finland	ERS2672902	PRJEB28342	Smeds et al. 2019
W69	Grey wolf	Finland	ERS2672903	PRJEB28342	Smeds et al. 2019
W7	Grey wolf	Finland	ERS2672904	PRJEB28342	Smeds et al. 2019
W70	Grey wolf	Finland	ERS2672905	PRJEB28342	Smeds et al. 2019
W71	Grey wolf	Finland	ERS2672906	PRJEB28342	Smeds et al. 2019
W72	Grey wolf	Finland	ERS2672907	PRJEB28342	Smeds et al. 2019
W73	Grey wolf	Finland	ERS2672908	PRJEB28342	Smeds et al. 2019

W74	Grey wolf	Finland	ERS2672909	PRJEB28342	Smeds et al. 2019
W75	Grey wolf	Finland	ERS2672910	PRJEB28342	Smeds et al. 2019
W76	Grey wolf	Finland	ERS2672911	PRJEB28342	Smeds et al. 2019
W77	Grey wolf	Finland	ERS2672912	PRJEB28342	Smeds et al. 2019
W78	Grey wolf	Finland	ERS2672913	PRJEB28342	Smeds et al. 2019
W79	Grey wolf	Finland	ERS2672914	PRJEB28342	Smeds et al. 2019
W8	Grey wolf	Finland	ERS2672915	PRJEB28342	Smeds et al. 2019
W80	Grey wolf	Finland	ERS2672916	PRJEB28342	Smeds et al. 2019
W81	Grey wolf	Finland	ERS2672917	PRJEB28342	Smeds et al. 2019
W82	Grey wolf	Finland	ERS2672918	PRJEB28342	Smeds et al. 2019
W83	Grey wolf	Finland	ERS2672919	PRJEB28342	Smeds et al. 2019
W84	Grey wolf	Finland	ERS2672920	PRJEB28342	Smeds et al. 2019
W85	Grey wolf	Finland	ERS2672921	PRJEB28342	Smeds et al. 2019
W86	Grey wolf	Finland	ERS2672922	PRJEB28342	Smeds et al. 2019
W87	Grey wolf	Finland	ERS2672923	PRJEB28342	Smeds et al. 2019
W88	Grey wolf	Finland	ERS2672924	PRJEB28342	Smeds et al. 2019
W89*	Grey wolf	Finland	ERS2672925	PRJEB28342	Smeds et al. 2019
W9*	Grey wolf	Finland	ERS2672926	PRJEB28342	Smeds et al. 2019
W90	Grey wolf	Finland	ERS2672927	PRJEB28342	Smeds et al. 2019
W91	Grey wolf	Finland	ERS2672928	PRJEB28342	Smeds et al. 2019
W92	Grey wolf	Finland	ERS2672929	PRJEB28342	Smeds et al. 2019
W93	Grey wolf	Finland	ERS2672930	PRJEB28342	Smeds et al. 2019
W94	Grey wolf	Finland	ERS2672931	PRJEB28342	Smeds et al. 2019
W95	Grey wolf	Finland	ERS2672932	PRJEB28342	Smeds et al. 2019
W96	Grey wolf	Finland	ERS2672933	PRJEB28342	Smeds et al. 2019
W97	Grey wolf	Finland	ERS2672934	PRJEB28342	Smeds et al. 2019
W98	Grey wolf	Finland	ERS2672935	PRJEB28342	Smeds et al. 2019
W99*	Grey wolf	Finland	ERS2672936	PRJEB28342	Smeds et al. 2019
W16	Grey wolf	Finland	ERS4802877	PRJEB39198	This study
W18	Grey wolf	Finland	ERS4802878	PRJEB39198	This study
W23	Grey wolf	Finland	ERS4802879	PRJEB39198	This study
W28	Grey wolf	Finland	ERS4802880	PRJEB39198	This study
W29	Grey wolf	Finland	ERS4802881	PRJEB39198	This study
W3	Grey wolf	Finland	ERS4802882	PRJEB39198	This study
W30	Grey wolf	Finland	ERS4802883	PRJEB39198	This study
W32	Grey wolf	Finland	ERS4802884	PRJEB39198	This study
W33	Grey wolf	Finland	ERS4802885	PRJEB39198	This study
W37	Grey wolf	Finland	ERS4802886	PRJEB39198	This study
W4	Grey wolf	Finland	ERS4802887	PRJEB39198	This study
W40	Grey wolf	Finland	ERS4802888	PRJEB39198	This study
W42	Grey wolf	Finland	ERS4802889	PRJEB39198	This study
W43	Grey wolf	Finland	ERS4802890	PRJEB39198	This study
W45	Grey wolf	Finland	ERS4802891	PRJEB39198	This study
W47	Grey wolf	Finland	ERS4802892	PRJEB39198	This study
W48	Grey wolf	Finland	ERS4802893	PRJEB39198	This study
W49	Grey wolf	Finland	ERS4802894	PRJEB39198	This study
W54	Grey wolf	Finland	ERS4802895	PRJEB39198	This study
W55	Grey wolf	Finland	ERS4802896	PRJEB39198	This study
W56	Grey wolf	Finland	ERS4802897	PRJEB39198	This study
W61	Grey wolf	Finland	ERS4802898	PRJEB39198	This study
W62	Grey wolf	Finland	ERS4802899	PRJEB39198	This study

V113	Grey wolf	Russia (Karelia)	ERS4802900	PRJEB39198	This study
V114	Grey wolf	Russia (Karelia)	ERS4802901	PRJEB39198	This study
V115	Grey wolf	Russia (Karelia)	ERS4802902	PRJEB39198	This study
V116	Grey wolf	Russia (Karelia)	ERS4802903	PRJEB39198	This study
V117	Grey wolf	Russia (Karelia)	ERS4802904	PRJEB39198	This study
V119	Grey wolf	Russia (Karelia)	ERS4802905	PRJEB39198	This study
V120	Grey wolf	Russia (Karelia)	ERS4802906	PRJEB39198	This study
V126	Grey wolf	Russia (Karelia)	ERS4802907	PRJEB39198	This study
V132	Grey wolf	Russia (Karelia)	ERS4802908	PRJEB39198	This study
V134	Grey wolf	Russia (Karelia)	ERS4802909	PRJEB39198	This study
V136	Grey wolf	Russia (Karelia)	ERS4802910	PRJEB39198	This study
V138	Grey wolf	Russia (Karelia)	ERS4802911	PRJEB39198	This study
V141	Grey wolf	Russia (Karelia)	ERS4802912	PRJEB39198	This study
V142	Grey wolf	Russia (Karelia)	ERS4802913	PRJEB39198	This study
V143	Grey wolf	Russia (Karelia)	ERS4802914	PRJEB39198	This study
V3064	Wolf x dog hybrid	Scandinavia	ERS4802915	PRJEB39198	This study
V3065	Wolf x dog hybrid	Scandinavia	ERS4802916	PRJEB39198	This study
V3069	Wolf x dog hybrid	Scandinavia	ERS4802917	PRJEB39198	This study
WoRu01	Grey wolf	Russia (Altai)	SRS402038	PRJNA448733	Wang et al. 2013, Plassais et al. 2019
WoRu02	Grey wolf	Russia (Chukotka)	SRS402061	PRJNA448733	Wang et al. 2013, Plassais et al. 2019
WoRu03	Grey wolf	Russia (Bryansk)	SRS402066	PRJNA448733	Wang et al. 2013, Plassais et al. 2019
WoNu01	Grey wolf	Arctic North America	SRS4196870	PRJNA512209	Robinson et al. 2019
WoNu02	Grey wolf	Arctic North America	SRS4196869	PRJNA512209	Robinson et al. 2019
WoNu03	Grey wolf	Arctic North America	SRS4196868	PRJNA512209	Robinson et al. 2019
WoNu04	Grey wolf	Arctic North America	SRS4196867	PRJNA512209	Robinson et al. 2019
WoAr01	Grey wolf	Arctic North America	SRS4647037	PRJNA532581	Phung et al. 2019
WoAr02	Grey wolf	Arctic North America	SRS4647036	PRJNA532581	Phung et al. 2019
WoAr03	Grey wolf	Arctic North America	SRS4647040	PRJNA532581	Phung et al. 2019
WoAr04	Grey wolf	Arctic North America	SRS4647038	PRJNA532581	Phung et al. 2019
WoAr05	Grey wolf	Arctic North America	SRS4647041	PRJNA532581	Phung et al. 2019
WoAr06	Grey wolf	Arctic North America	SRS4647039	PRJNA532581	Phung et al. 2019
WoMo01	Grey wolf	China	SRS3887566	PRJNA494719	vonHoldt et al. 2017
WoQi01	Grey wolf	China	SRS3887562	PRJNA494719	vonHoldt et al. 2017
WoCh04	Grey wolf	China	SRS401789	PRJNA448733	Wang et al. 2013, Plassais et al. 2019
WoCh05	Grey wolf	China	SRS1135628	PRJNA266585	Bai et al. 2015
WoCh06	Grey wolf	China	SRS1135629	PRJNA266585	Bai et al. 2015
WoCh07	Grey wolf	China	SRS1135626	PRJNA266585	Bai et al. 2015
WoCh08	Grey wolf	China	SRS1135623	PRJNA266585	Bai et al. 2015
WoCh09	Grey wolf	China	SRS1135627	PRJNA266585	Bai et al. 2015
WoCh10	Grey wolf	China	SRS1135622	PRJNA266585	Bai et al. 2015

WoCh11	Grey wolf	China	SRS1135621	PRJNA266585	Bai et al. 2015
AlMa01	Arctic/Nordic dog	Alaskan malamute	ERS1755464	PRJEB16012	
AlMa02	Arctic/Nordic dog	Alaskan malamute	SRS3254041	PRJNA448733	Plassais et al. 2019
AlMa03	Arctic/Nordic dog	Alaskan malamute	SRS1129580	PRJNA448733	Plassais et al. 2019
Chin01	Arctic/Nordic dog	Chinook	SRS932169	PRJNA448733	Plassais et al. 2019
FiLa01	Arctic/Nordic dog	Finnish lapphund	SRS1129832	PRJNA448733	Plassais et al. 2019
GrDo01	Arctic/Nordic dog	Greenland dog	SRS1129835	PRJNA448733	Plassais et al. 2019
Jamt01	Arctic/Nordic dog	Jamthund	SRS1135625	PRJNA266585	Bai et al. 2015
LaHe01	Arctic/Nordic dog	Lapponian herder	SRS1129836	PRJNA448733	Plassais et al. 2019
Lund01	Arctic/Nordic dog	Lundehund	SRS386919	PRJNA448733	Plassais et al. 2019
Newf01	Arctic/Nordic dog	Newfoundland	SRS5354855	PRJNA263947	
NoEl01	Arctic/Nordic dog	Norwegian elkhound	SRS1129834	PRJNA266585	Bai et al. 2015
NoEl02	Arctic/Nordic dog	Norwegian elkhound	SRS3259289	PRJNA448733	Plassais et al. 2019
Samo01	Arctic/Nordic dog	Samoyed	SRS3259307	PRJNA448733	Plassais et al. 2019
Samo02	Arctic/Nordic dog	Samoyed	SRS1135616	PRJNA448733	Plassais et al. 2019
SiHu01	Arctic/Nordic dog	Siberian husky	ERS1755452	PRJEB16012	
SiHu02	Arctic/Nordic dog	Siberian husky	SRS1135615	PRJNA448733	Plassais et al. 2019
SiHu03	Arctic/Nordic dog	Siberian husky	SRS984799	PRJNA288568	
SwLa01	Arctic/Nordic dog	Swedish lapphund	SRS1135613	PRJNA448733	Plassais et al. 2019
ChCr01	Asian dog	Chinese crest	SRS661484	PRJNA448733	Plassais et al. 2019
ChVi01	Asian dog	Chinese village dog	SRS1397564	PRJNA448733	Plassais et al. 2019
ChVi02	Asian dog	Chinese village dog	SRS1135601	PRJNA448733	Plassais et al. 2019
ChVi03	Asian dog	Chinese village dog	SRS1135605	PRJNA448733	Plassais et al. 2019
ChVi04	Asian dog	Chinese village dog	SRS1135609	PRJNA448733	Plassais et al. 2019
DiIn01	Asian dog	Diqing indigenous dog	SRS540596	PRJNA448733	Plassais et al. 2019
DiIn02	Asian dog	Diqing indigenous dog	SRS540597	PRJNA448733	Plassais et al. 2019
InVi01	Asian dog	Indian village dog	SRS520065	PRJNA448733	Plassais et al. 2019
InVi02	Asian dog	Indian village dog	SRS520061	PRJNA448733	Plassais et al. 2019
JiDo01	Asian dog	Jindo dog	DRS001112	PRJNA448733	Plassais et al. 2019
KuDo01	Asian dog	Kunming dog	SRS540528	PRJNA448733	Plassais et al. 2019
KuDo02	Asian dog	Kunming dog	SRS540531	PRJNA448733	Plassais et al. 2019
LiIn01	Asian dog	Lijiang indigenous dog	SRS540591	PRJNA448733	Plassais et al. 2019
LiIn02	Asian dog	Lijiang indigenous dog	SRS540568	PRJNA448733	Plassais et al. 2019
ShIn01	Asian dog	Shiba Inu	SRS1124450	PRJNA448733	Plassais et al. 2019
ShPe01	Asian dog	Shar pei	SRS1539504	PRJNA448733	Plassais et al. 2019
ShPe02	Asian dog	Shar pei	SRS1539503	PRJNA448733	Plassais et al. 2019
ShTz01	Asian dog	Shih tzu	SRS3259310	PRJNA448733	Plassais et al. 2019
TiMa01	Asian dog	Tibetan mastiff	SRS540631	PRJNA448733	Plassais et al. 2019
TiMa02	Asian dog	Tibetan mastiff	SRS540625	PRJNA448733	Plassais et al. 2019
TiTe01	Asian dog	Tibetan terrier	SRS932145	PRJNA448733	Plassais et al. 2019
TiTe02	Asian dog	Tibetan terrier	SRS932148	PRJNA448733	Plassais et al. 2019
ViVi01	Asian dog	Vietnamese village dog	SRS1135631	PRJNA448733	Plassais et al. 2019
ViVi02	Asian dog	Vietnamese village dog	SRS1135632	PRJNA448733	Plassais et al. 2019
ViVi03	Asian dog	Vietnamese village dog	SRS1135633	PRJNA448733	Plassais et al. 2019
YiIn01	Asian dog	Yingjiang	SRS540536	PRJNA448733	Plassais et al. 2019

		indigenous dog			
YiIn02	Asian dog	Yingjiang indigenous dog	SRS540534	PRJNA448733	Plassais et al. 2019
AiTe01	European dog	Airdale terrier	SRS1124461	PRJNA448733	Plassais et al. 2019
AiTe02	European dog	Airdale terrier	SRS932170	PRJNA448733	Plassais et al. 2019
BaHo01	European dog	Bavarian hound	ERS1755455	PRJEB16012	
Bass01	European dog	Basset	ERS1408667	PRJNA448733	Plassais et al. 2019
Beag01	European dog	Beagle	SRS1867899	PRJNA448733	Plassais et al. 2019
BeMa01	European dog	Belgian malinois	SRS402079	PRJNA448733	Plassais et al. 2019
BePi01	European dog	Berger picard	SRS1867896	PRJNA448733	Plassais et al. 2019
BeSh01	European dog	Belgian sheepdog	SRS3259211	PRJNA448733	Plassais et al. 2019
BoCo01	European dog	Border collie	ERS1789008	PRJEB16012	
BoCo02	European dog	Border collie	ERS1789005	PRJEB16012	
BoFl01	European dog	Bouvier des Flandres	SRS1867889	PRJNA448733	Plassais et al. 2019
BoTe01	European dog	Border terrier	SRS1867891	PRJNA448733	Plassais et al. 2019
BoTe02	European dog	Border terrier	SRS1124455	PRJNA448733	Plassais et al. 2019
Boxe01	European dog	Boxer	SRS661481	PRJNA448733	Plassais et al. 2019
BuMa01	European dog	Bull mastiff	ERS1638210	PRJEB16012	
BuTe01	European dog	Bull terrier	SRS3259249	PRJNA448733	Plassais et al. 2019
BuTe02	European dog	Bull terrier	SRS3259247	PRJNA448733	Plassais et al. 2019
CoSp01	European dog	Cocker spaniel	ERS1406079	PRJNA448733	Plassais et al. 2019
Dach01	European dog	Dachshund	ERS1755460	PRJEB16012	
Dach02	European dog	Dachshund	SRS1867894	PRJNA448733	Plassais et al. 2019
Dobe01	European dog	Dobermann	SRS932142	PRJNA448733	Plassais et al. 2019
EnCo01	European dog	English cocker spaniel	SRS661482	PRJNA448733	Plassais et al. 2019
EnSe01	European dog	English setter	SRS1867900	PRJNA448733	Plassais et al. 2019
EnSe02	European dog	English setter	SRS1124453	PRJNA448733	Plassais et al. 2019
EnSS01	European dog	English springer spaniel	SRS1124454	PRJNA448733	Plassais et al. 2019
EnSS02	European dog	English springer spaniel	SRS932160	PRJNA448733	Plassais et al. 2019
FiSp01	European dog	Field spaniel	SRS1867886	PRJNA448733	Plassais et al. 2019
GeSh01	European dog	German shepard	ERS1535185	PRJNA448733	Plassais et al. 2019
GeSh02	European dog	German shepard	SRS932159	PRJNA448733	Plassais et al. 2019
GHTe01	European dog	German hunting terrier	ERS1789013	PRJEB16012	
GoRe01	European dog	Golden retriever	SRS3259257	PRJNA448733	Plassais et al. 2019
GoRe02	European dog	Golden retriever	ERS1681145	PRJEB16012	
GrDa01	European dog	Great dane	ERS1755450	PRJEB16012	
GrDa02	European dog	Great dane	SRS1867890	PRJNA448733	Plassais et al. 2019
GSMD01	European dog	Greater Swiss mountain dog	SRS3259263	PRJNA448733	Plassais et al. 2019
GSMD02	European dog	Greater Swiss mountain dog	SRS1867892	PRJNA448733	Plassais et al. 2019
IrTe01	European dog	Irish terrier	SRS1867902	PRJNA448733	Plassais et al. 2019
IrWS01	European dog	Irish water spaniel	SRS3259266	PRJNA448733	Plassais et al. 2019
JRTe01	European dog	Jack Russell terrier	ERS1789012	PRJEB16012	
JRTe02	European dog	Jack Russell terrier	SRS1124459	PRJNA448733	Plassais et al. 2019
Komo01	European dog	Komondor	SRS3259269	PRJNA448733	Plassais et al. 2019
LaRe01	European dog	Labrador retriever	SRS3259279	PRJNA448733	Plassais et al. 2019
LaRe02	European dog	Labrador retriever	SRS3259278	PRJNA448733	Plassais et al. 2019
LaRo01	European dog	Lagotto Romagnolo	ERS1408669	PRJNA448733	Plassais et al. 2019

Lowc01	European dog	Lowchen	SRS1124448	PRJNA448733	Plassais et al. 2019
Mali01	European dog	Malinois	ERS1789014	PRJNA448733	Plassais et al. 2019
MiBu01	European dog	Miniature bullterrier	ERS1406076	PRJNA448733	Plassais et al. 2019
PoPo01	European dog	Portuguese podengo	SRS932163	PRJNA448733	Plassais et al. 2019
PoWa01	European dog	Portugese water dog	SRS3259297	PRJNA448733	Plassais et al. 2019
PoWa02	European dog	Portugese water dog	SRS3259295	PRJNA448733	Plassais et al. 2019
PWCo01	European dog	Pembroke Welsh corgi	SRS732549	PRJNA448733	Plassais et al. 2019
Rott01	European dog	Rottweiler	SRS3259302	PRJNA448733	Plassais et al. 2019
Rott02	European dog	Rottweiler	SRS3259301	PRJNA448733	Plassais et al. 2019
SaBe01	European dog	Saint Bernard	SRS932165	PRJNA448733	Plassais et al. 2019
ScDe01	European dog	Scottish deerhound	SRS932150	PRJNA448733	Plassais et al. 2019
ScTe01	European dog	Scottish terrier	SRS3259308	PRJNA448733	Plassais et al. 2019
ShSh01	European dog	Shetland sheepdog	SRS932138	PRJNA448733	Plassais et al. 2019
StPo01	European dog	Standard poodle	SRS932143	PRJNA448733	Plassais et al. 2019
StPo02	European dog	Standard poodle	SRS932171	PRJNA448733	Plassais et al. 2019
StSc01	European dog	Standard schnauzer	SRS1867905	PRJNA448733	Plassais et al. 2019
StSc02	European dog	Standard schnauzer	SRS1867887	PRJNA448733	Plassais et al. 2019
Weim01	European dog	Weimaraner	ERS1406081	PRJNA448733	Plassais et al. 2019
WHWT01	European dog	West highland white terrier	ERS1408668	PRJNA448733	Plassais et al. 2019
WHWT02	European dog	West highland white terrier	ERS1406080	PRJNA448733	Plassais et al. 2019
YoTe01	European dog	Yorkshire terrier	SRS3259389	PRJNA448733	Plassais et al. 2019
YoTe02	European dog	Yorkshire terrier	SRS3259346	PRJNA448733	Plassais et al. 2019

* Sampled in Finland but born in Scandinavia.

Table S2. Statistics from PCADMIX output for the three wolf-dog F_1 hybrids using different window sizes. The last two columns show proportions of the genome assigned to only dog or only wolf ancestry.

Window size	Individual	#Switches/Mb	Only dog ancestry	Only wolf ancestry
20 SNPs	V3064	0.271	0.0122	0.0451
	V3065	0.265	0.0109	0.0425
	V3069	0.262	0.0116	0.0421
50 SNPs	V3064	0.249	0.0158	0.0353
	V3065	0.250	0.0131	0.0322
	V3069	0.243	0.0137	0.0315
100 SNPs	V3064	0.234	0.0194	0.0312
	V3065	0.231	0.0149	0.0240
	V3069	0.218	0.0154	0.0263
200 SNPs	V3064	0.211	0.0268	0.0276
	V3065	0.208	0.0212	0.0160
	V3069	0.205	0.0245	0.0211

Supplementary figures

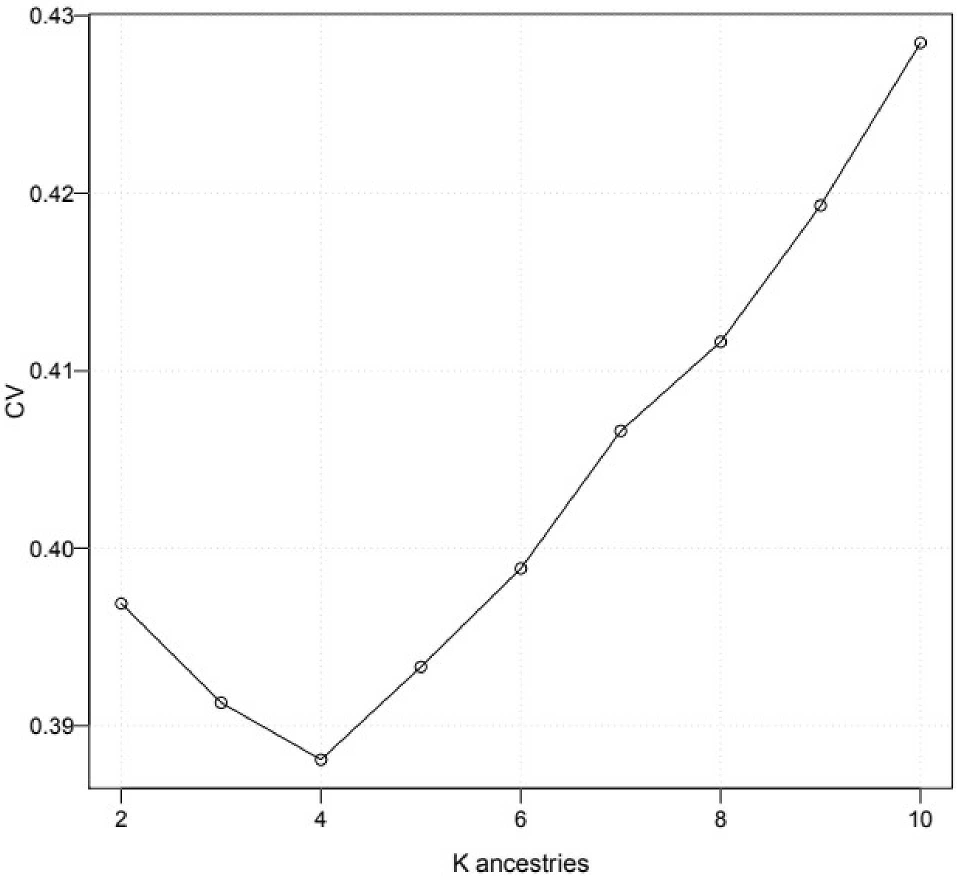

Figure S1. Cross-validation errors from ADMIXTURE runs with K=2-10.

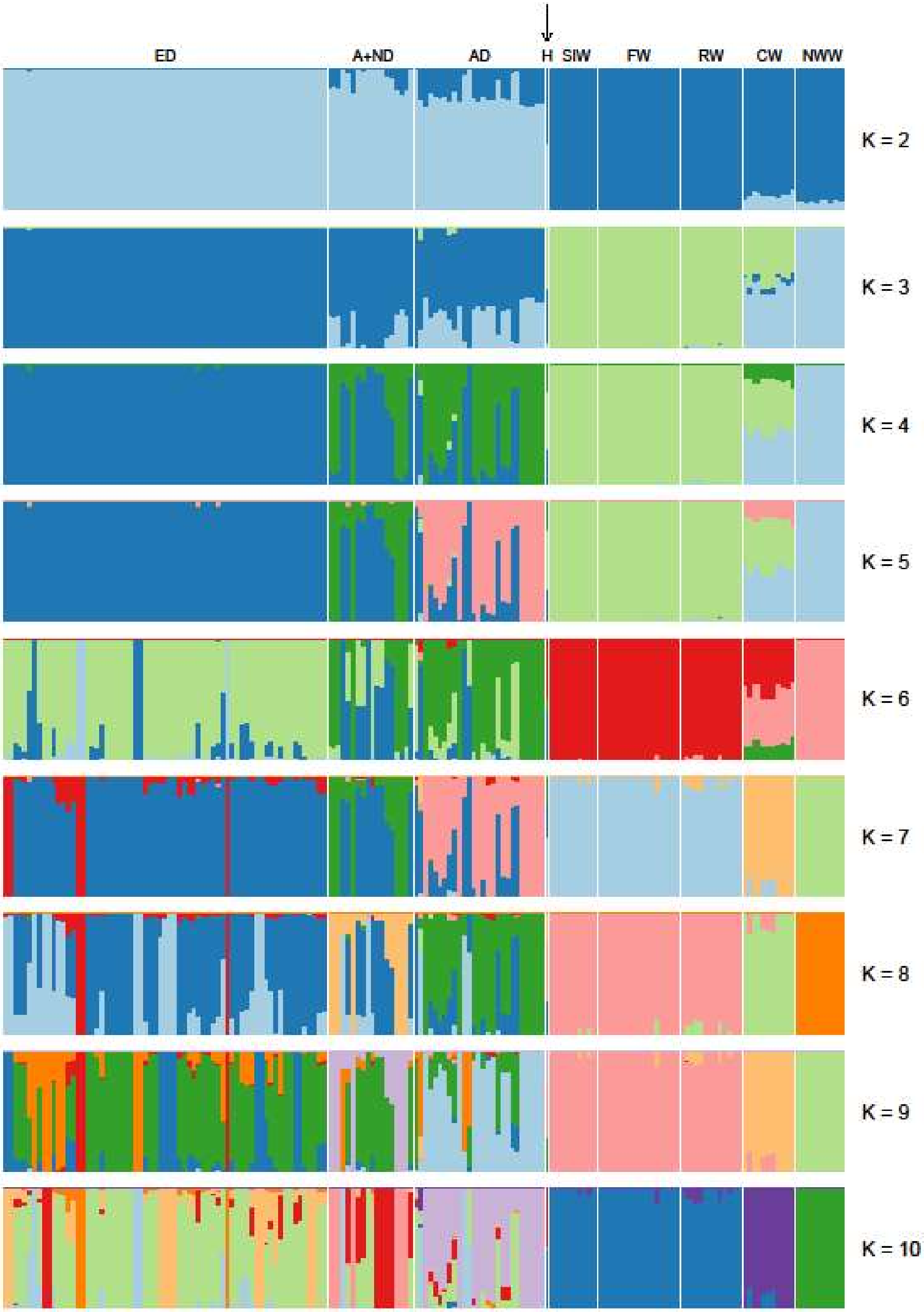

Figure S2. Result from ADMIXTURE using K=2-10 clusters. This is an extension of Figure 3. Abbreviations: ED – European dogs, A+ND – Arctic and Nordic dogs, AD – Asian dogs, H – F_1 hybrid, SIW – Scandinavian immigrant wolves, FW – Finnish wolves, RW – Russian Karelian wolves, CW – Chinese wolves, NWW – New World wolves. The F_1 hybrid is marked with an arrow for visibility.

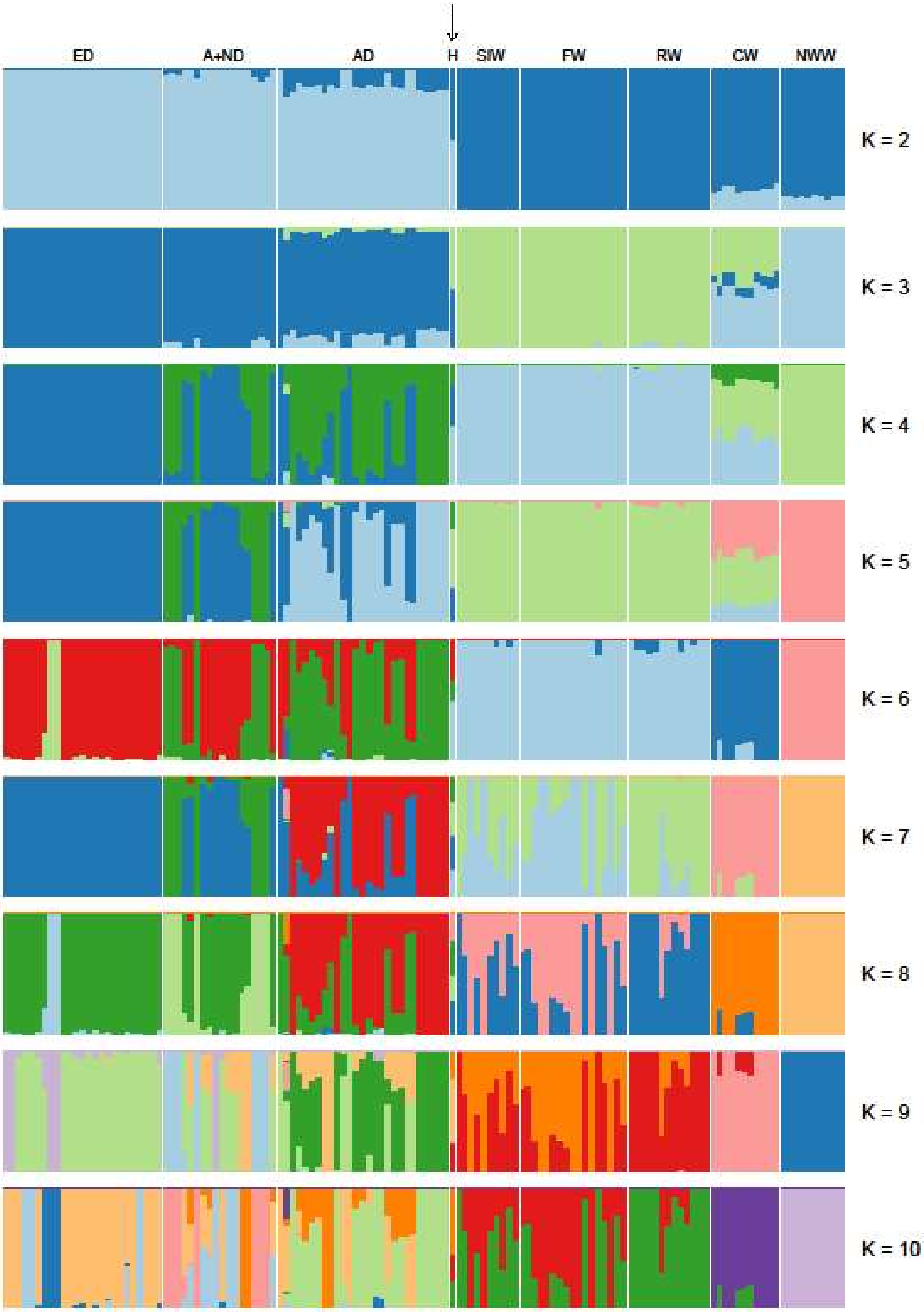

Figure S3. Same as Figure S2 but with the group “European Dogs” down-sampled to avoid sample size bias. Abbreviations as in Figure S2.

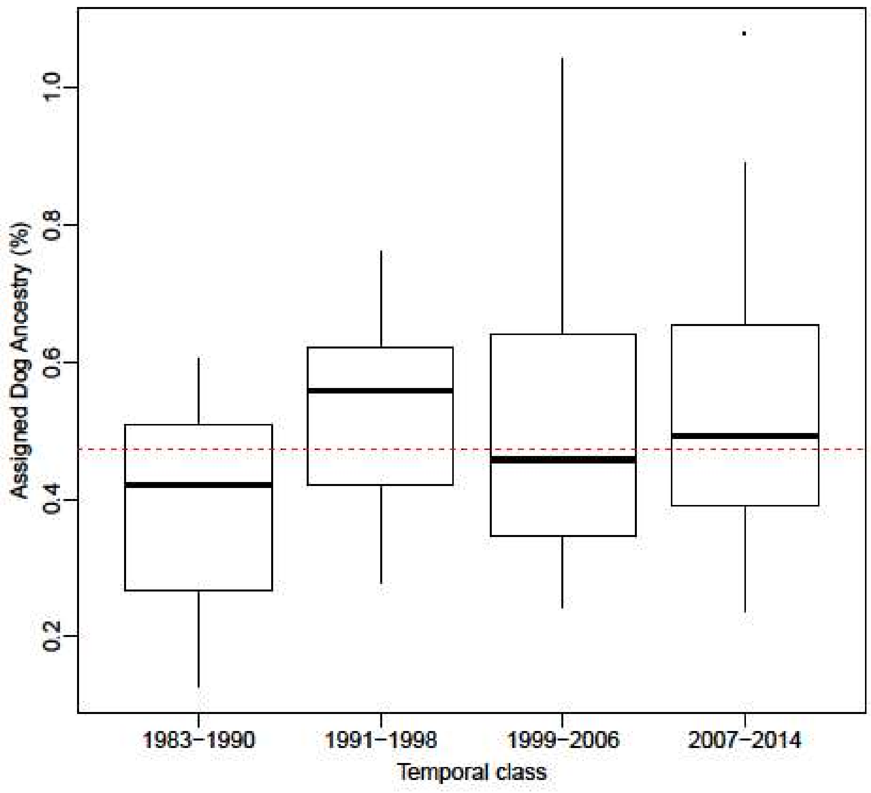

Figure S4. Mixed ancestry assigned by PCADMIX for Scandinavian wolves divided into different temporal classes. The red dashed line indicates assigned dog ancestry in the female founder. The differences among groups are not statistically significant (ANOVA, $p = 0.12$).

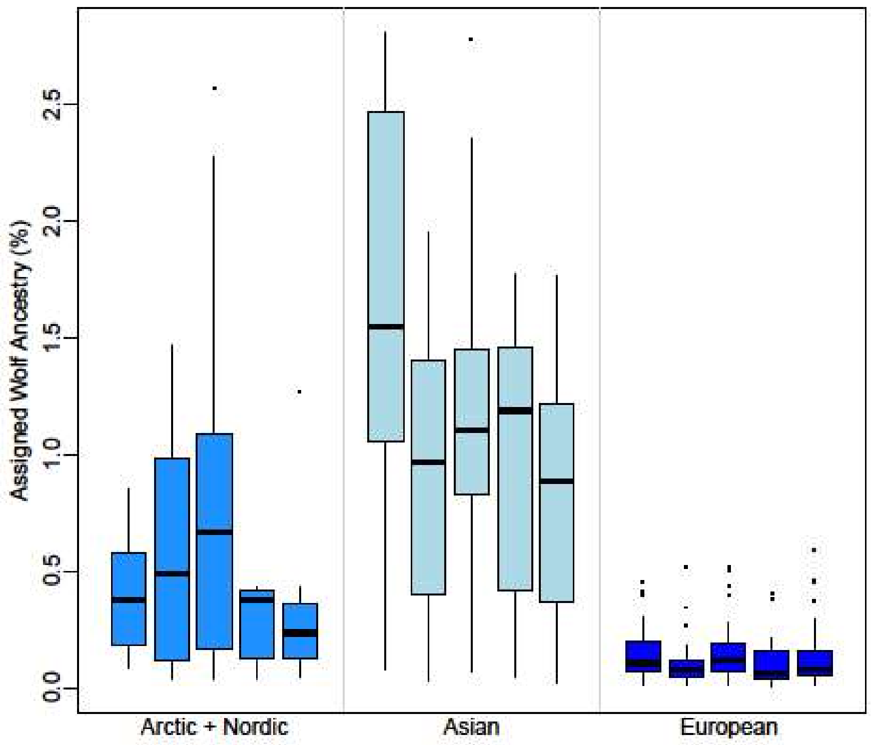

Figure S5. Mixed ancestry in different groups of dogs assigned by PCADMIX. Five different runs were performed with a random set of 50 dogs and 50 wolves used for the reference ancestral groups, while the remaining dogs were used to assess wolf ancestry.

(a)

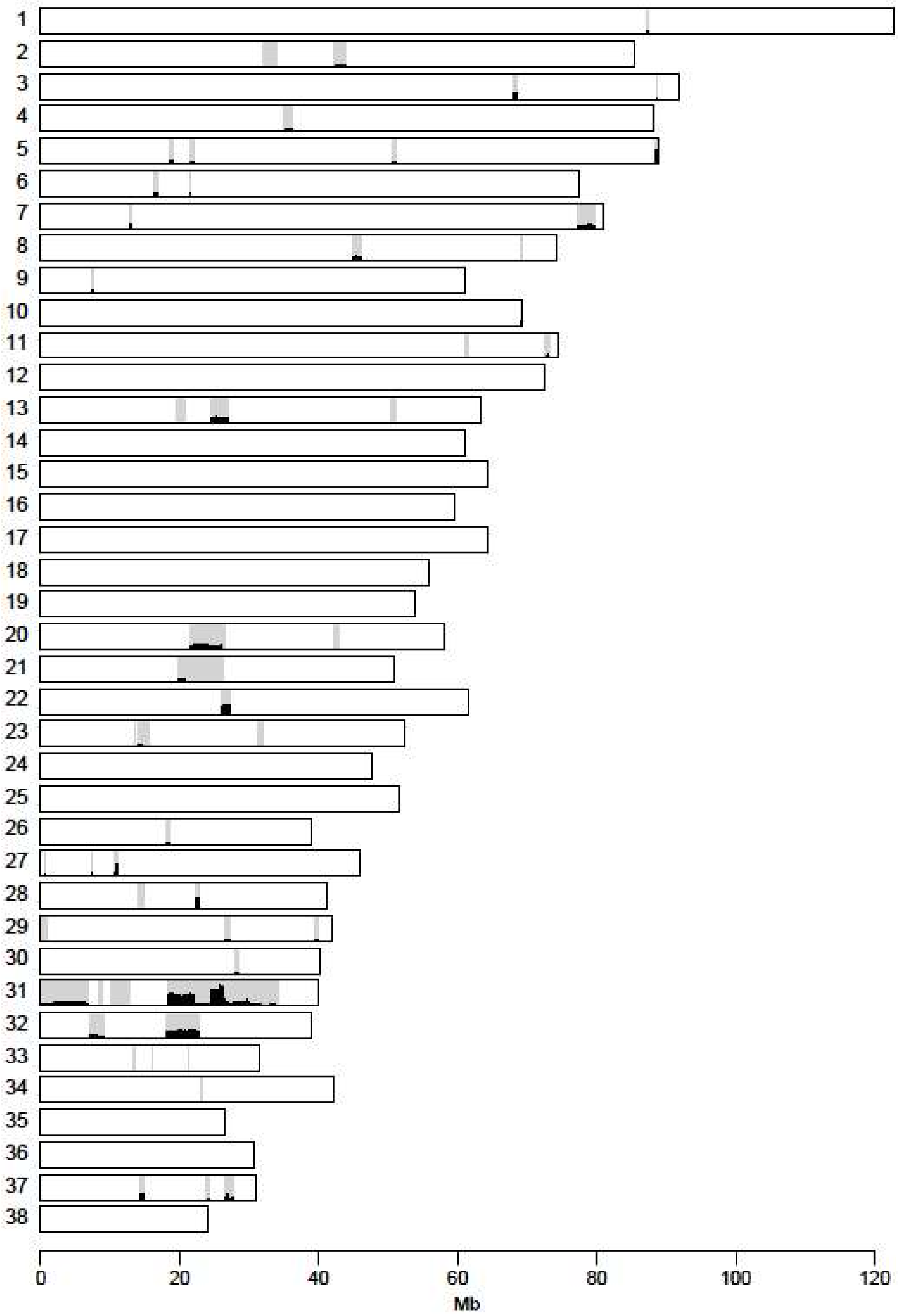
1
2
3
4
5
6
7
8
9
10
11
12
13
14
15
16
17
18
19
20
21
22
23
24
25
26
27
28
29
30
31
32
33
34
35
36
37
38
0
20
40
60
80
100
120
Mb

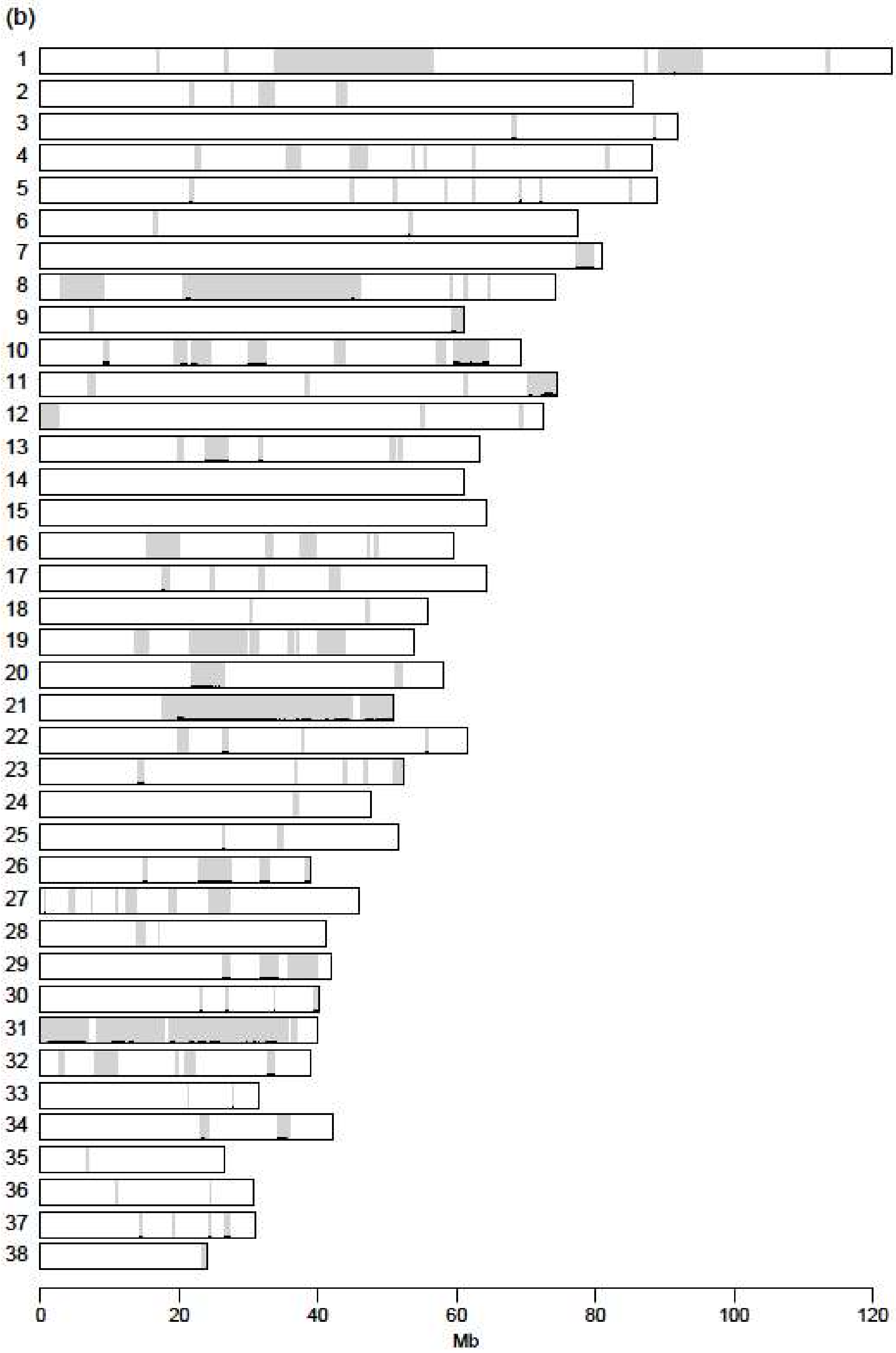
(b)
1
2
3
4
5
6
7
8
9
10
11
12
13
14
15
16
17
18
19
20
21
22
23
24
25
26
27
28
29
30
31
32
33
34
35
36
37
38
0
20
40
60
80
100
120
Mb

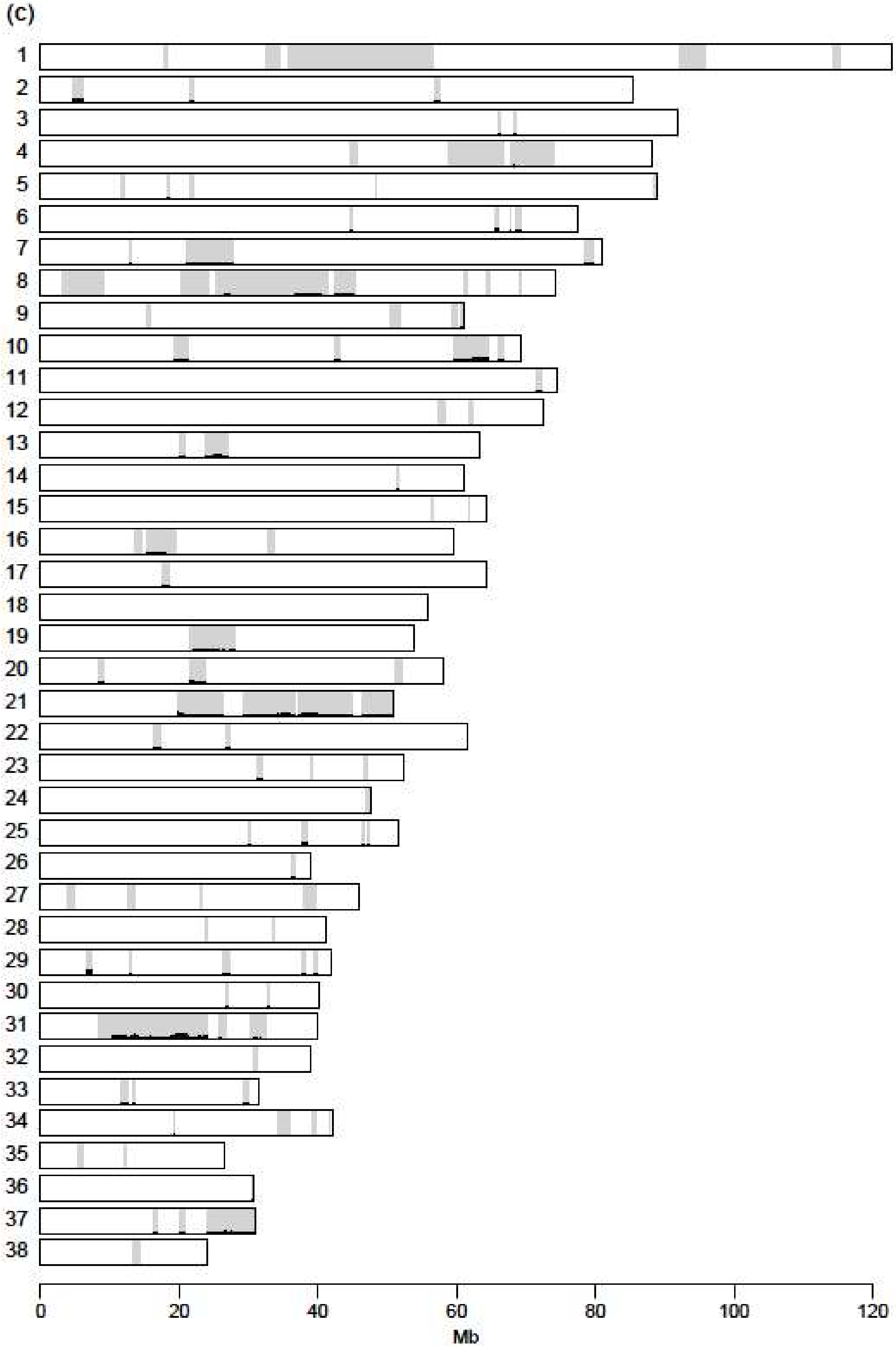

Figure S6. Summary of dog ancestry blocks from PCADMIX for all individuals in different wolf populations. For each chromosome, grey background indicates that the region has dog ancestry in at least one individual. The y-axis goes from 0 to 1 and black bars denote the fraction of individual chromosomes in the population that show dog ancestry in a certain window. For example, if a region has a black bar of half the total height, it means half of the chromosomes in the population

have assigned dog ancestry in this region. (a) Scandinavian, (b) Finnish, and (c) Russian Karelian wolves.

Supplementary references

Bai, B., Zhao, W., Tang, B., Wang, Y., Wang, L., Zhang, Z., . . . Zhang, Y. (2015). DoGSD: the dog and wolf genome SNP database. *Nucleic Acids Research*, *43*(D1), D777–D783.

Kardos, M., Åkesson, M., Fountain, T., Flagstad, Ø., Liberg, O., Olason, P., . . . Ellegren, H. (2018). Genomic consequences of intensive inbreeding in an isolated wolf population. *Nature Ecology & Evolution, 2*(1), 124-131.

Phung T. N., Wayne R. K., Wilson M. A. and Lohmueller K. E. (2019) Complex patterns of sex-biased demography in canines. *Proceedings of the Royal Society of London. Series B: Biological Sciences 286*(1976).

Plassais, J., Kim, J., Davis, B.W., Karyadi, D. M., Hogan, A. N., Harris, A. C., . . . Ostrander, E. A. (2019) Whole genome sequencing of canids reveals genomic regions under selection and variants influencing morphology. *Nature Communications*, *10*(1489).

Robinson, J. A., Räikkönen, J., Vucetich, L. M., Vucetich, J. A., Peterson, R. O., Lohmueller, K. E. and Wayne, R. K. (2019). Genomic signatures of extensive inbreeding in Isle Royale wolves, a population on the threshold of extinction. *Science Advances*, *5*(5).

Smeds, L., Kojola, I., & Ellegren, H. (2019). The evolutionary history of grey wolf Y chromosomes. *Molecular Ecology, 28*(9), 2173-2191.

vonHoldt, B., Fan, Z., Ortega-Del Vecchyo, D., Wayne R. K. (2017). EPAS1 variants in high altitude Tibetan wolves were selectively introgressed into highland dogs. *PeerJ*, 5 e3522.

Wang, G., Zhai, W., Yang, H., Fan, R., Cao, X., Zhong, L., . . . Zhang, Y. (2013) The genomics of selection in dogs and the parallel evolution between dogs and humans. *Nature Communications*, *4*(1860).
